# Autism-like social deficit generated by *Dock4* deficiency is rescued by restoration of Rac1 activity and NMDA receptor function

**DOI:** 10.1038/s41380-019-0472-7

**Published:** 2019-08-06

**Authors:** Daji Guo, Yinghui Peng, Laijian Wang, Xiaoyu Sun, Xiaojun Wang, Chunmei Liang, Xiaoman Yang, Shengnan Li, Junyu Xu, Wen-Cai Ye, Bin Jiang, Lei Shi

**Affiliations:** 1 JNU-HKUST Joint Laboratory for Neuroscience and Innovative Drug Research, College of Pharmacy, Jinan University, Guangzhou, 510632 Guangdong China; 2 Guangdong Province Key Laboratory of Brain Function and Disease, Zhongshan School of Medicine, Sun Yat-sen University, Guangzhou, 510080 Guangdong China; 3 Department of Neurobiology, Key Laboratory of Medical Neurobiology of Ministry of Health, Zhejiang Province Key Laboratory of Neurobiology, Zhejiang University School of Medicine, Hangzhou, 310058 Zhejiang China

**Keywords:** Neuroscience, Autism spectrum disorders

## Abstract

Genetic studies of autism spectrum disorder (ASD) have revealed multigene variations that converge on synaptic dysfunction. *DOCK4*, a gene at 7q31.1 that encodes the Rac1 guanine nucleotide exchange factor Dock4, has been identified as a risk gene for ASD and other neuropsychiatric disorders. However, whether and how Dock4 disruption leads to ASD features through a synaptic mechanism remain unexplored. We generated and characterized a line of *Dock4* knockout (KO) mice, which intriguingly displayed a series of ASD-like behaviors, including impaired social novelty preference, abnormal isolation-induced pup vocalizations, elevated anxiety, and perturbed object and spatial learning. Mice with conditional deletion of *Dock4* in hippocampal CA1 recapitulated social preference deficit in KO mice. Examination in CA1 pyramidal neurons revealed that excitatory synaptic transmission was drastically attenuated in KO mice, accompanied by decreased spine density and synaptic content of AMPA (α-amino-3-hydroxy-5-methyl-4-isoxazolepropionic acid)- and NMDA (*N*-methyl-_D_-aspartate)-type glutamate receptors. Moreover, *Dock4* deficiency markedly reduced Rac1 activity in the hippocampus, which resulted in downregulation of global protein synthesis and diminished expression of AMPA and NMDA receptor subunits. Notably, Rac1 replenishment in the hippocampal CA1 of *Dock4* KO mice restored excitatory synaptic transmission and corrected impaired social deficits in these mice, and pharmacological activation of NMDA receptors also restored social novelty preference in *Dock4* KO mice. Together, our findings uncover a previously unrecognized Dock4-Rac1-dependent mechanism involved in regulating hippocampal excitatory synaptic transmission and social behavior.

## Introduction

Autism spectrum disorder (ASD) is a complex neurodevelopmental disorder characterized by the core symptoms of social deficits, language communication failure, and stereotyped behaviors. ASD exhibits a strong genetic basis, and increasing evidence from inherited and de novo gene variations suggests a notable convergence on synapse pathophysiology in ASD, particularly dysfunction of excitatory synaptic transmission [1, 2]. Rac1, a master actin regulator, plays critical roles in modulating both the localization of neurotransmitter receptors and the architecture of excitatory synapses [3]. Rac1 has been identified as a converging factor downstream of numerous proteins encoded by high-risk ASD genes, such as *SHANK* [4–6], *FMR1* (fragile X mental retardation-1) [7, 8], *NRXN* [9], and *AUTS2* [10]. Intriguingly, dysfunctions of specific genes might produce opposite effects on Rac1 activity. For instance, *SHANK* and *AUTS2* dysfunctions lead to repressed Rac1 activity, whereas *FMR1* dysfunction leads to excessive Rac1 activity. Moreover, both excessive and insufficient Rac1 activity induced by missense mutations in *RAC1* itself cause developmental disorders presenting diverse phenotypes [11]. These findings suggest that tightly regulated Rac1 activity is crucial for ASD-related neuronal function. Supporting this notion, restoration of normal activity of Rac1 or its downstream effectors was demonstrated as a successful approach for correcting autism-like behaviors and synaptic abnormalities [12–15]. Therefore, delineating the pattern of Rac1 regulation at synapses could be critical for elucidating the synaptic basis of specific behaviors associated with ASD.

Rac1 activity is tightly controlled by Rac1 guanine nucleotide exchange factors (GEFs) and GTPase-activating proteins (GAPs), two protein families that directly activate and inhibit Rac1, respectively [16]. Dock4, a member of the Dock (dedicator of cytokinesis) family, is an atypical Rac1 GEF linked to ASD and other neuropsychiatric disorders [17]. *DOCK4* is located at chromosome band 7q31.1 [18, 19], an autism-susceptible locus resided by several ASD-associated genes involved in development and language regulation. Recent studies have identified a number of *DOCK4* variations associated with ASD, including both single nucleotide variations (SNVs) and chromosome microdeletions or duplications (Supplementary Table 1) [18-26]. Many of these variations are believed to result in loss-of-function of *DOCK4*. For instance, several microdeletions lead to loss of various lengths of the C-terminal portion of Dock4 protein [19, 21, 26], which is responsible for its Rac1-activating ability [27]. Moreover, evidence has shown decreased mRNA levels of *DOCK4* in blood derived lymphoblastoid cells of two populations of ASD patients (Supplementary Fig. 1) [28]. Dock4 has been found to plays critical roles in axon guidance, dendritic development, and dendritic spine formation, but these results were obtained from in vitro preparations [27, 29–31]. Whether and how Dock4 dysfunction leads to synaptic impairments and ASD-like behavioral abnormalities remain unexplored.

To investigate the role of Dock4 in ASD pathophysiology, we generated a mouse line lacking whole-body Dock4 expression (*Dock4* KO mice). *Dock4* KO mice displayed abnormalities in social behavior, vocalizations, anxiety levels, and learning and memory, accompanied by reduced hippocampal excitatory synapse number and transmission. Conditional deletion of *Dock4* in hippocampal CA1 led to similar social deficit observed in the KO mice. Notably, *Dock4* deficiency markedly decreased Rac1 activity in the hippocampus, which resulted in downregulated global protein synthesis and reduced expression of AMPAR (α-amino-3-hydroxy-5-methyl-4-isoxazolepropionic acid receptor) and NMDAR (*N*-methyl-_D_-aspartate receptor) subunits. Lentiviral expression of Rac1 in hippocampal CA1 of *Dock4* KO mice restored excitatory synaptic transmission and corrected impaired social behaviors in these mice, and NMDAR activation also restored social behaviors in KO mice. Together, our findings reveal a previously undescribed Dock4-Rac1-dependent mechanism underlying the protein synthesis of glutamate receptors, which is crucial for excitatory synaptic transmission and social behavior.

## Materials and methods

### Generation of *Dock4* knockout (KO) mice

*Dock4* KO mice (C57BL/6 background) were generated using a standard strategy of Cre-LoxP recombination (Biocytogen). The targeting vector contained Exon 3 of a *Dock4* homology region, covering 5.4 kb upstream and 4.2 kb downstream of Exon 3. An FRT-flanked Neo cassette was inserted 3′ of Exon 3, and two LoxP sites were introduced 5′ of Exon 3 and 3′ of Neo, respectively. F1 mice carrying the Neo-floxed *Dock4* allele (*Dock4*^fl-neo/+^) were crossed with Flp mice (C57BL/6 background) to remove the Neo cassette to obtain *Dock4* floxed mice (*Dock4*^fl/fl^ mice). Alternatively, *Dock4*^fl-neo/+^ mice were crossed with EIIa-Cre germline deleter mice (C57BL/6 background) to obtain *Dock4*^+/+^ (wild-type; WT), *Dock4*^+/−^ (heterozygous; HET) and *Dock4*^−/−^ (KO) mice. Because of the deletion and the resulting frameshift, the null allele generates a 61-aa truncated protein (41 aa of Dock4 N-terminal sequence and 20 aa of frame-shifted nonsense sequence) instead of the full-length 1978 aa protein (Supplementary Figs. 2 and 3, Supplementary methods).

### Behavioral tests

*Dock4* KO, HET, and WT littermates of both genders, aged 3–6 months old, were used for all behavioral analyses except pup-retrieval assay. In pup-retrieval assay, only virgin female KO, HET, and WT littermates were used. Moreover, female *Dock4* HET and KO mice displaying stereotyped behavior were tested in open field test and were excluded in the other tests. For virus injection and drug treatment studies, animals were randomly assigned to two groups, each according to gender (male: female ratio is roughly 1:1) and body weight. Investigators were blind to genotype or treatment of the mice being tested during all experiments. The detailed protocols of behavioral tests are presented in Supplementary Information.

### Antibodies and reagents

The primary antibodies used in Western blot analysis were purchased from the following commercial suppliers: Dock4 (ab85723, 1:1000) and PSD95 (ab2723, 1:1000) were from Abcam; GluA1 (PC246, 1:1000), GluA2 (MAB397, 1:1000), GluN2A (AB1555P, 1:1000), GluN2B (AB1557P and 06-000, 1:1000), Rac1 (05-389, 1:2000), and puromycin (MABE343, 1:10000) were from Merck Millipore; GluN1 (32-0500, 1:1000) was from Invitrogen; Dock4 (WH0009732M1, 1:1000), ɑ-tubulin (T6199, 1:5000), and Flag (F1804, 1:1000) were from Sigma; GAPDH (A01020, 1:5000) was from Abbkine. The secondary antibodies for Western blot were purchased from Cell Signaling Technology (anti-mouse IgG-HRP, 7076s; anti-Rb IgG-HRP, 7074s). Pharmacological inhibitors MG132 and NSC23766 were purchased from Selleck. Puromycin was purchased from Merck Millipore. cDNAs of Dock4 and Rac1 and their mutants, and Dock4 shRNA were described previously [27, 32]. Neuro-2a cells were purchased from ATCC and were routinely tested for mycoplasma contamination-free before use.

### Statistics

For all behavioral tests (except nesting scoring), multiple groups were compared using one-way or two-way analysis of variance (ANOVA) with Bonferroni’s or Dunn’s Multiple Comparison Test, and two groups were compared using unpaired *t* test. To evaluate the nesting score, comparisons were performed using Kruskal-Wallis test with Dunn’s Multiple Comparison Test. Detailed statistical analysis of all behavioral studies are shown in Supplementary Tables 2 and 3. For electrophysiological analyses, statistical significance was assessed using unpaired *t* test, one-way or two-way ANOVA, or two-sample Kolmogorov-Smirnov test. Western blotting results were quantified through densitometric measurement of each band (or all bands for puromycin blots) by using Quantity One 1-D Analysis Software (Bio-Rad). Statistically significant differences between two groups and multiple groups were determined using paired or unpaired *t* tests and ANOVA followed by Bonferroni’s Multiple Comparison Test, respectively. Data are expressed as means ± SEM (standard error of the mean). Sample size chosen for each study was established by previous pilot and published studies. For electrophysiological study and biological characterizations using animal tissues, each group included half male and half female. All statistical analyses were performed using GraphPad Prism software; normal distribution and similar variance within each comparison group of data were checked before any parametric analyses; *P* < 0.05 was considered significant.

All experimental procedures involving the use of animals were approved by the Ethics Committee on Animal Experiments at Jinan University and Sun Yat-sen University, and were strictly performed according to the National Institutes of Health guidelines of the Care and Use of Laboratory Animals. All efforts were made to minimize the suffering and the number of animals used.

Additional details of materials and methods are shown in Supplementary Information.

## Results

### *Dock4* KO mice exhibit social deficits

**Fig. 1:**
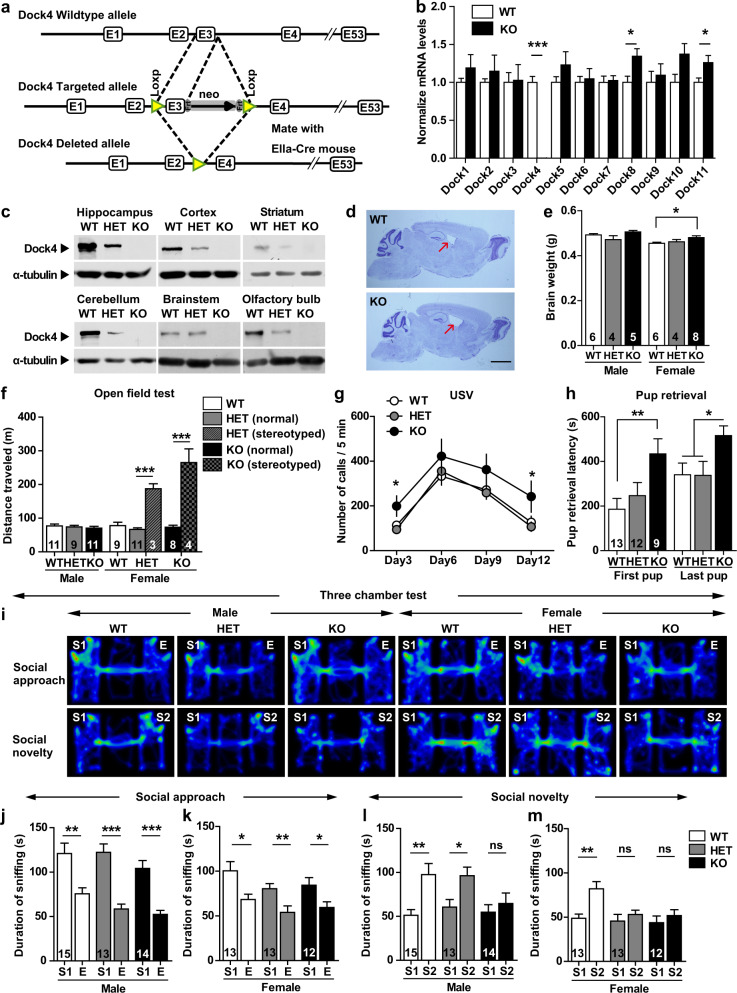
*Dock4* KO mice exhibit altered social behavior. **a** Strategy used for generating *Dock4* KO mice. **b** Brain mRNA levels of *Dock4* and other Dock-family members were assessed using quantitative RT-PCR; mRNA levels of KO mice were normalized to those of WT mice. *n* = 6 WT or KO mice; *P < 0.05, ***P < 0.001, unpaired *t* test. **c** Dock4 protein expression in different brain regions of WT, HET, and KO mice; α-tubulin was used as a loading control. **d** Nissl staining of sagittal brain sections from 5-month-old WT and KO mice. Brain lateral ventricles were slightly enlarged in KO mice (arrows). Scale bar, 2 mm. **e** Brain weight measurements from 5-month-old WT, HET, and KO mice. *P < 0.05, one-way ANOVA with Bonferroni’s Multiple Comparison Test. **f** Distance traveled by WT, HET, and KO mice in open-field test. Hyperactivity was displayed by a small population of HET (~1.7%; 3/172) and KO (~9.1%; 4/44) female mice that exhibited stereotyped circling behavior. ***P < 0.001, unpaired *t* test. **g** Ultrasonic vocalizations of pups at P3, P6, P9, and P12 during maternal separation for 5 min. *n* = 28, 28, 29, and 26, respectively for WT; *n* = 39, 41, 38, and 38, respectively for HET; *n* = 13, 15, 15, and 13, respectively for KO. *P < 0.05, one-way ANOVA with Bonferroni’s Multiple Comparison Test. **h** Pup-retrieval assay was performed to evaluate maternal behavior of virgin female mice. Latency of retrieving the first pup and all 3 pups was measured in a 10-min trial. *P < 0.05, **P < 0.01, one-way ANOVA with Bonferroni’s Multiple Comparison Test. **i** Representative heatmap of movement of male and female WT, HET, and KO mice during social approach and social novelty phases of the Three-chamber test. **j**, **k** Duration spent in sniffing different cups, measured for male (**j**) and female (**k**) WT, HET, and KO mice during social approach. **l**, **m** Duration spent in sniffing different cups, measured for male (**l**) and female (**m**) WT, HET, and KO mice during social novelty preference. E, empty cup; S1, cup containing stranger mouse #1; S2, cup containing stranger mouse #2. *P < 0.05, **P < 0.01, ***P < 0.001, ns, no significant, unpaired *t* test. *n* values of each group are displayed on the corresponding bars of the bar charts. Error bars: SEM

A *Dock4* KO mouse line was established, and the loss of *Dock4* expression in the mice was verified using quantitative real-time PCR (qRT-PCR) and western blotting (Fig. 1a, Supplementary Figs. 2 and 3). The *Dock4* transcript was completely absent in the KO brain and reduced by approximately half in the HET brain (Fig. 1b, Supplementary Fig. 3d). Transcripts of 10 other Dock-family members, Dock 1–3 and 5–11, did not vary extensively (Fig. 1b). In accord with previous observations [27], Dock4 was found to be expressed in various brain regions and highly enriched in the hippocampus (Fig. 1c). No Dock4 was detected in any brain region or other organs in KO mice, whereas reduced amounts of Dock4 were found in the corresponding HET tissues (Fig. 1c, Supplementary Fig. 3f).

Despite showing slightly lower natality (Supplementary Fig. 4a), *Dock4* HET and KO mice exhibited similar growth rate (weight gain; Supplementary Fig. 4b, c), ingestion, and mating as WT mice did. Brain architecture in *Dock4* KO mice was grossly normal, with slight enlargement of the lateral ventricles (Fig. 1d, Supplementary Fig. 4d–g), and the brain was slightly heavier in female KO mice than in female WT mice (Fig. 1e). Notably, a small population of female HET (1.7%) and KO (9.1%) mice showed autism-like hyperactivity and stereotyped behavior, such as continual circling in the home cage and open field (Fig. 1f, Supplementary Fig. 5a, Supplementary Videos 1–3). The male HET and KO mice and the majority of the female HET and KO mice displayed mostly normal locomotion in the open field (Fig. 1f) and did not show stereotyped behaviors (Supplementary Fig. 5b, c). This suggests that *Dock4* deficiency might contribute to a risk factor of stereotyped behavior particularly in females. Because the number of stereotyped mice was small, these mice were excluded in the subsequent studies.

To investigate language communication in *Dock4* KO mice, we evaluated ultrasonic vocalizations in pups when isolated from their dam within the first two weeks after birth. Interestingly, *Dock4* KO pups emitted more calls at postnatal days 3, 6, 9, and 12 than their WT littermates (Fig. 1g). The total call duration was also longer in KO pups than in WT pups, but the average peak frequency of all calls was similar in both genotypes (Supplementary Fig. 6a, b). This altered vocal behavior was reminiscent of some ASD mice models [33–35], suggesting abnormal communication in *Dock4* KO mice. We next performed tests to assess several features of the social behavior. First, the ability of nesting, a nature in mice that tightly correlates with social behaviors such as parenting and reproduction, was evaluated. As a result, mice of all genotypes performed equally well in nest building (Supplementary Fig. 6c–e). Second, maternal behavior was measured in a pup-retrieval assay by using virgin female mice. Relative to WT littermates, KO females spent significantly longer time to retrieve the pups (Fig. 1h), suggesting poorer maternal behavior of KO females. Finally, social approach and social preference behaviors were assessed using the Three-chamber test [36]. In the social approach phase, WT, HET, and KO mice all performed normally, spending more time exploring the stranger conspecific than the object (Fig. 1i–k; Supplementary Fig. 6f, g). However, in the social novelty phase, both male and female KO mice and HET female mice failed to distinguish between familiar and newly introduced unfamiliar conspecifics (Fig. 1i, l, m; Supplementary Fig. 6h, i). Together, characterizations of the ASD core symptoms revealed that *Dock4* KO mice exhibited both language and social deficits, and displayed increased tendency of stereotyped behavior.

### Elevated anxiety and defective learning and memory in *Dock4* KO mice

**Fig. 2:**
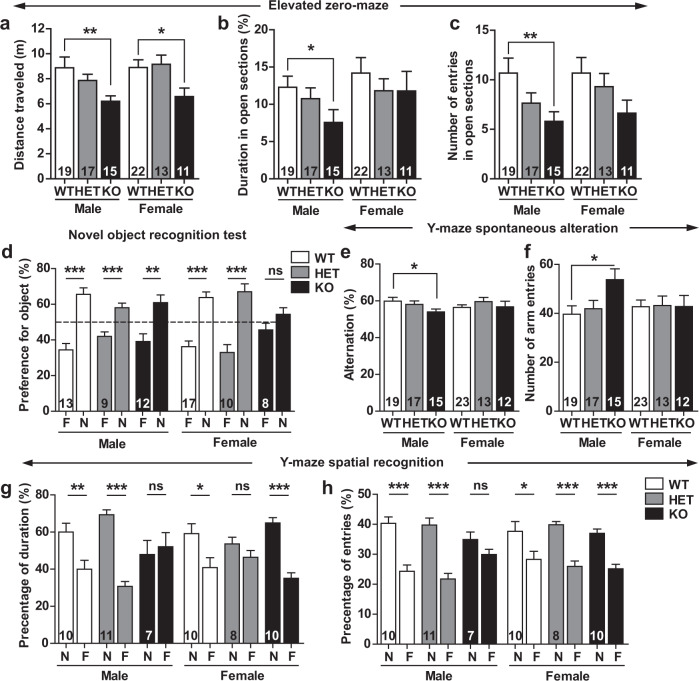
*Dock4* KO mice display elevated anxiety and impaired learning and memory. **a**–**c** WT, HET, and KO mice were tested in an elevated zero-maze, and the following variables were measured and analyzed: (**a**) Total distance traveled; (**b**) duration (% of total time) spent in open sections; and (**c**) number of entries into open sections. *P < 0.05, **P < 0.01, one-way ANOVA with Bonferroni’s Multiple Comparison Test. (**d**) Novel object recognition memory of WT, HET, and KO mice was examined at 24 h after training. Female KO mice showed aberrant recognition memory. F, familiar object; N, novel object. ***P < 0.001, ns, no significant, unpaired *t* test. **e**, **f** Alternation (**e**) and number (**f**) of arm entries of WT, HET, and KO mice during 8 min of free exploration in Y-maze test. ***P < 0.001, unpaired *t* test. **g, h** Duration (**g**) and number of entries (**h**) in novel or familiar arm, measured for WT, HET, and KO mice in 5-min trials in Y-maze. F, familiar arm; N, novel arm. *P < 0.05, **P < 0.01, ***P < 0.001, ns, no significant, unpaired *t* test. *n* values of each group are displayed on the corresponding bars of the bar charts. Error bars: SEM

Besides the core symptoms, ASD subjects commonly present co-occurring symptoms that include anxiety and intellectual disability [37, 38]. We measured anxiety behavior in elevated zero-maze. Relative to WT mice, *Dock4* KO mice traveled a markedly shortened total distance (Fig. 2a). Moreover, the anxiety levels were higher in KO males than KO females: the KO males not only spent less time in the open sections of the maze (Fig. 2b), but also entered the open area less frequently (Fig. 2c). Furthermore, the KO males exhibited reduced mobility in both the open and closed sections, as indicated by the decreased travel distance and velocity (Supplementary Fig. 7a–d). To measure cognitive ability, mice were subjected to several learning and memory tasks, including novel object recognition test, Y-maze test, and Morris water-maze test. In the novel object recognition task, female KO mice failed to distinguish the novel object from the familiar one (Fig. 2d). In the Y-maze task, working memory was tested by measuring the alternate exploring behavior among the three arms of the maze: KO males displayed decreased alternation, and their arm-entry numbers were significantly higher than those of WT males (Fig. 2e, f), which suggests defective working memory in KO males. In a distinct Y-maze paradigm, spatial recognition memory between familiar and novel arms was tested. KO males failed to distinguish between the two arms, suggesting that KO males show aberrant spatial recognition (Fig. 2g, h). In the Morris water-maze task, WT, HET, and KO mice displayed similar latency in locating the submerged platform over a 7-day training period (Supplementary Fig. 7e–h). Collectively, these results indicate that *Dock4* KO mice manifest all three core domains of ASD symptoms, and display elevated anxiety and impaired object and spatial learning (Supplementary Table 4).

### *Dock4* deficiency in hippocampus leads to social deficit and impaired excitatory synaptic transmission

**Fig. 3:**
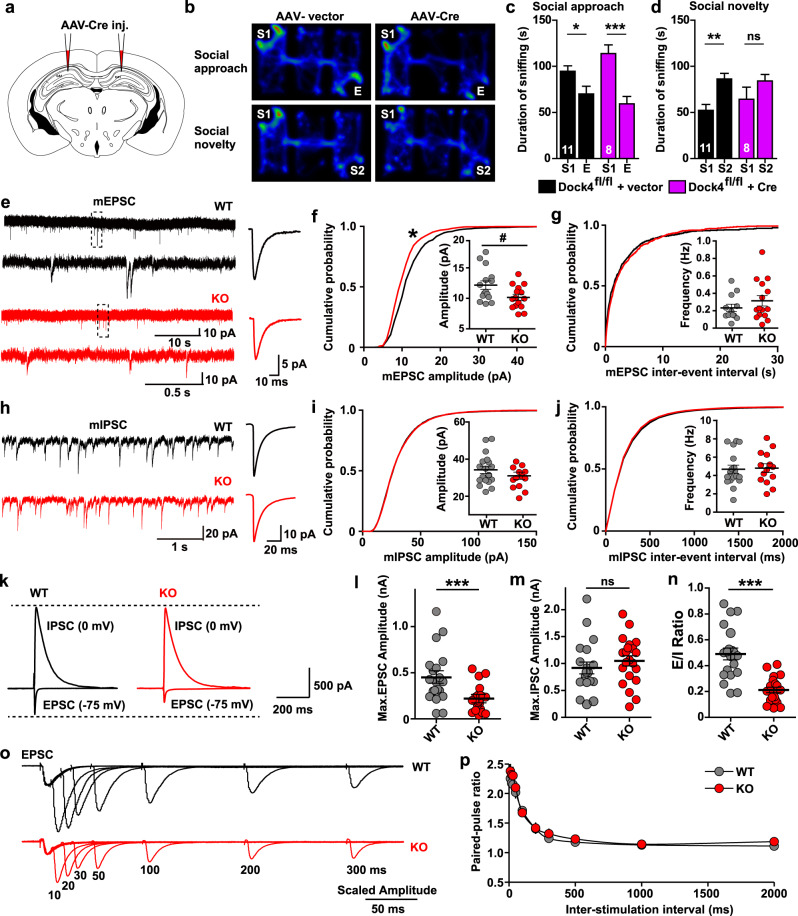
Dock4 deficiency in hippocampus leads to social deficit and impaired excitatory synaptic transmission. **a** Adeno-associated virus (AAV)-Cre was bilaterally injected into the hippocampal CA1 region of *Dock4*^fl/fl^ mice. **b** Representative heatmap of movement of *Dock4*^fl/fl^ + AAV-vector or *Dock4*^fl/fl^ + AAV-Cre mice in the Three-chamber test at 4 weeks after virus injection. **c**, **d** Duration spent by two groups of mice in sniffing different cups during social approach (**c**) and social novelty (**d**) phases. E, empty cup; S1, cup containing stranger mouse #1; S2, cup containing stranger mouse #2. *n* = 11 *Dock4*^fl/fl^ + AAV-vector mice (6 males and 5 females), and *n* = 8 *Dock4*^fl/fl^ + AAV-Cre mice (4 males and 4 females). *P < 0.05, **P < 0.01, ***P < 0.001, ns, no significant, unpaired *t* test. **e** Representative (left) and average (right) traces of mEPSCs recorded from WT (black) and KO (red) hippocampal CA1 pyramidal cells. Magnified traces from the boxed regions are shown below. **f**, **g** Cumulative probability of mEPSC amplitude (**f**) and inter-event interval (**g**) in WT (15 cells, 4 mice) and KO (15 cells, 3 mice) groups. Insets: average mEPSC amplitude (**f**) and frequency (**g**) from WT and KO groups *P < 0.05, two sample Kolmogorov-Smirnov test. ^#^*P* < 0.05, unpaired *t* test. **h** Representative (left) and average (right) *t*races of mIPSCs recorded from WT (black) and KO (red) hippocampal CA1 pyramidal cells. **i**, **j** Cumulative probability of mIPSC amplitude (**i**) and inter-event interval (**j**) in WT (18 cells, 3 mice) and KO (15 cells, 3 mice) groups. Insets: average mIPSC amplitude (**i**) and frequency (**j**) from WT and KO groups. **k** Representative traces of evoked EPSC and IPSC in same CA1 pyramidal cells from WT (black) and KO (red) mice. **l**, **m** The maximal EPSC (**l**) and IPSC (**m**) recorded from WT (20 cells, 6 mice) and KO (21 cells, 5 mice) groups. ***P < 0.001, ns, not significant, unpaired *t* test. (**n**) The calculated E/I ratio from WT and KO groups. ***P < 0.001, unpaired *t* test. **o** Representative traces of paired-pulse ratio (PPR) of EPSCs at different inter-stimulus intervals recorded from WT (black) and KO (red) mice. **p** The plot of PPR vs. different inter-stimulus intervals (WT: 10 cells, 4 mice; KO: 10 cells, 4 mice). Error bars: SEM

The hippocampus, in which Dock4 is highly expressed, plays a central role in integrating brain networks for social and recognition memories [39]. To investigate the effect of Dock4 in hippocampus, we specifically deleted Dock4 in hippocampus by adeno-associated virus (AAV)-mediated delivery of Cre into hippocampal CA1 region of *Dock4*^fl/fl^ mice (Fig. 3a, Supplementary Fig. 8a, b). Intriguingly, these hippocampal conditional KO mice failed to distinguish between familiar and newly introduced unfamiliar conspecifics in Three-chamber test, recapitulating the social deficit in whole-body KO mice (Fig. 3b–d, Supplementary Fig. 8c–e). This result indicates that social novelty preference requires Dock4-dependent regulation of hippocampal function. We then explored the synaptic basis in the hippocampus that underlies the aberrant social behavior in *Dock4* KO mice. The amplitude of miniature excitatory synaptic currents (mEPSCs) recorded in hippocampal CA1 pyramidal neurons was smaller in KO mice than in WT mice (Fig. 3e–g), whereas miniature inhibitory synaptic currents (mIPSCs) were unaltered in KO mice (Fig. 3h–j). To determine whether excitatory/inhibition (E/I) balance is affected by *Dock4* deficiency, we recorded both evoked EPSC and IPSC from same pyramidal cells. Notably, whereas IPSC showed no change, EPSC amplitude reduced remarkably in KO cells (Fig. 3k–m). As a result, the E/I ratio of KO cells was substantially smaller than that of WT cells (Fig. 3n), suggesting a shift of E/I balance toward inhibition in KO hippocampal CA1 pyramidal neurons. To examine whether the reduced EPSC in KO neurons is resulted from compromised presynaptic function, paired-pulse ratios (PPRs) of EPSCs were measured. Normal paired-pulse facilitation was observed in both WT and KO neurons (Fig. 3o, p), suggesting that the diminished excitatory synaptic transmission in *Dock4* KO hippocampus is probably attributed to postsynaptic dysfunctions.

### Disrupted AMPA and NMDA receptor functions in *Dock4* KO hippocampus

**Fig. 4:**
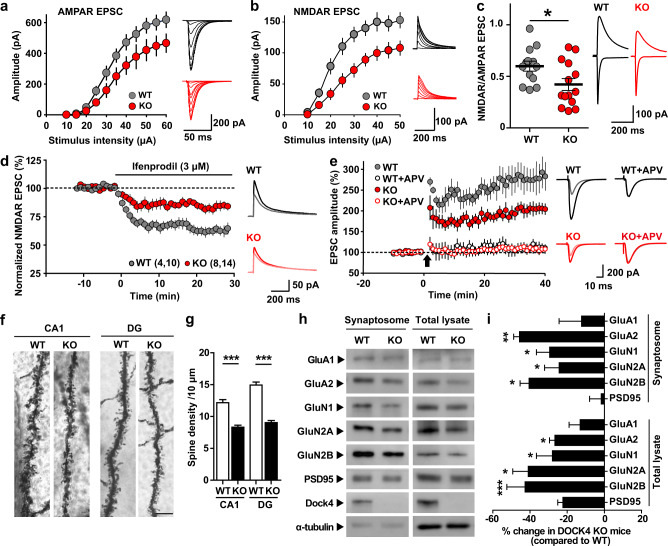
AMPAR and NMDAR-dependent synaptic transmission is impaired in *Dock4* KO hippocampus. **a**, **b** KO neurons displayed a downward input-output curve of both AMPAR-EPSC (**a**) (25–30% decrease at strongest stimulation relative to WT; *n* = 10 cells from 3 WT mice; *n* = 12 cells from 4 KO mice, *P* < 0.001, two-way AVOVA) and NMDAR-EPSC (**b**) (30–35% decrease at strongest stimulation relative to WT; *n* = 26 cells from 6 WT mice; *n* = 24 cells from 5 KO mice, *P* < 0.001, two-way AVOVA). Representative traces are shown in the right panel of each graph. **c** NMDAR-EPSC to AMPAR-EPSC ratios from WT (13 cells, 5 mice) and KO (15 cells, 5 mice) groups. Representative traces are shown on the right. *P < 0.05, unpaired *t* test. **d** KO mice showed a reduction in the proportion of synaptic GluN2B. Values are presented as change of NMDAR-EPSC (% of baseline) after application of ifenprodil, an GluN2B inhibitor. Representative traces before and at 20–30 min after application of ifenprodil are shown on the right. *n* = 10 cells from 4 WT mice; *n* = 14 cells from 8 KO mice. *P* < 0.05, unpaired *t* test. **e** Time-course changes of EPSC amplitude (% of baseline) after a pairing protocol (0 mV, 2 Hz, 360 pulses; arrow), with or without application of APV, measured from WT (Ctr, 12 cells, 5 mice; APV: 9 cells, 4 mice) and KO (Ctr: 14 cells, 6 mice, APV: 13 cells, 6 mice) groups. *P* = 0.0241, WT vs. KO; *P* = 0.00001, WT + APV vs. WT; *P* = 0.0001, KO + APV vs. KO, unpaired *t* test. Representative traces before and at 25–35 min after pairing are shown on the right. **f** Representative Golgi staining images of dendritic spines of hippocampal CA1 and dentate gyrus (DG) neurons in WT and KO mice. Scale bar, 5 μm. **g** Spine density was decreased in both the hippocampal CA1 and DG regions of KO mice. *n* = 3 WT and KO pairs. ***P < 0.001, unpaired *t* test. **h** Expression levels of AMPAR and NMDAR subunits in the synaptosomal fraction and total lysate of the hippocampus of WT and KO mice. GluA2, GluN1, GluN2A, and GluN2B subunits were decreased at the synapse as well as in the whole hippocampus. α-tubulin served as a loading control. **i** Alteration of synaptosomal and total expression of indicated proteins in the KO hippocampus, expressed as fold-change of that in WT. *n* = 4 WT and KO pairs, *P < 0.05, **P < 0.01, ***P < 0.001, unpaired *t* test. Error bars: SEM

To examine whether the excitatory postsynaptic function is impaired in KO hippocampus, the input-output properties of AMPAR- and NMDAR-mediated EPSCs were measured separately. Notably, the EPSCs mediated by both receptors were significantly decreased in KO cells after stimulation at a series of intensities, with the reduction of NMDAR-EPSCs being more prominent (Fig. 4a, b). Accordingly, the ratio of NMDAR-EPSCs to AMPAR-EPSCs was lowered by *Dock4* deficiency (Fig. 4c). We also measured the functional synaptic composition of NMDAR subunits by isolating NMDAR-EPSCs blocked by the GluN2B antagonist ifenprodil (3 μM); the blockage of NMDAR-EPSCs by ifenprodil was significantly weaker in KO neurons than in WT neurons, which suggests that KO neurons contain less synaptic proportion of GluN2B-containing receptors (Fig. 4d). Because NMDAR function is important for the induction of both long-term potentiation (LTP) and long-term depression (LTD), we examined whether these two forms of synaptic plasticity are altered by *Dock4* deficiency. Whole-cell recordings were used to examine LTP at Schaffer collateral-CA1 synapses induced by a pairing protocol, which revealed that LTP was significantly reduced in KO neurons as compared with that in WT neurons (Fig. 4e). Application of APV, an NMDAR antagonist, completely blocked the induction of LTP in either WT or KO neurons, suggesting that only NMDA-dependent LTP was induced using this protocol (Fig. 4e). On the other hand, LTD was induced either by low frequency stimulation or by application of NMDA. Whereas both forms of LTD were hardly induced at adult as reported [40], they are similarly induced in WT and KO hippocampus at young postnatal age, suggesting that LTD is intact in KO mice (Supplementary Fig. 9).

We next investigated whether the impairment of excitatory synaptic transmission was associated with altered synaptic number and expression of AMPARs and NMDARs. Dendrite arborization was normal in KO hippocampus (Supplementary Fig. 10), but KO neurons had significantly decreased dendritic spines in both CA1 and dentate gyrus (DG) when compared to WT neurons (Fig. 4f, g). Notably, the synaptic expression of several AMPAR and NMDAR subunits (including GluA2, GluN1, GluN2A, and GluN2B) was markedly reduced in the KO hippocampus (Fig. 4h, i). Therefore, the diminished AMPAR- and NMDAR-mediated EPSCs can potentially be attributed to the decreased synaptic content of the corresponding receptors. To further examine whether the reduction of these receptor subunits was limited to the synapse, we measured their global expression in the hippocampus. Intriguingly, GluA2, GluN1, GluN2A, and GluN2B still showed 10–30% reduction in expression (Fig. 4h, i). Reduction of some AMPAR and NMDAR subunits was also observed in prefrontal cortex, but not striatum and cerebellum of KO mice (Supplementary Fig. 11). In contrast to the protein level changes, the mRNA levels of these receptor subunits were unaltered (Supplementary Fig. 12). These results suggest that the receptor decrease at the protein level might be due to protein homeostasis regulation.

### Dock4 maintains normal protein synthesis of AMPAR and NMDAR subunits in a Rac1-dependent manner

**Fig. 5:**
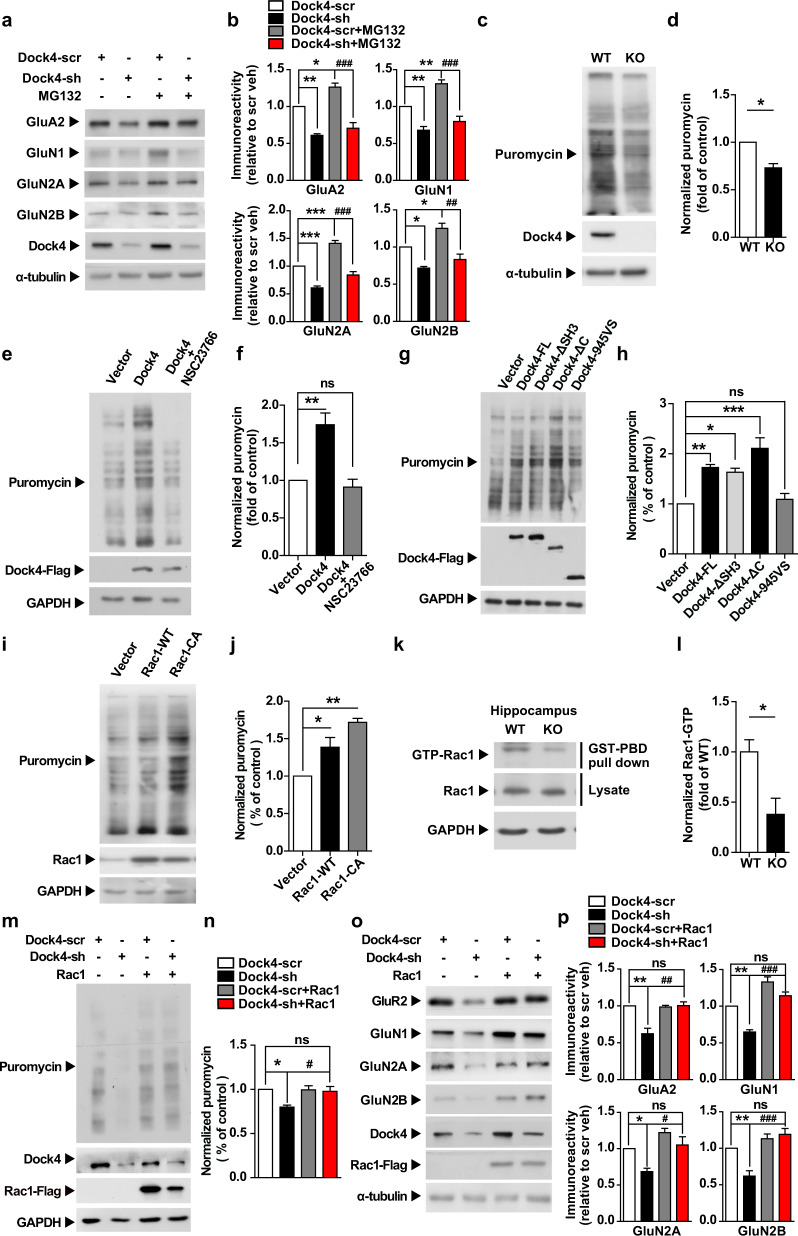
Dock4 maintains normal expression of glutamate receptor subunits in a Rac1-dependent manner. **a** Dock4 was knocked down in hippocampal neurons by using Dock4 shRNA (Dock4-sh) or a scrambled shRNA (Dock4-scr) as a control at 5 DIV, and the proteasome inhibitor MG132 (2 μM) was added 24 h before protein samples were collected at 9 DIV. **b** Expression level of each receptor subunit was quantified and normalized to Dock4-scr group. *P < 0.05, **P < 0.01, ***P < 0.001, ^###^*P* < 0.001, one-way ANOVA with Bonferroni’s Multiple Comparison Test from 3 independent experiments. **c** Hippocampal neurons from WT or KO littermates were treated with puromycin at 9 DIV, and the puromycin-labeled, newly synthesized polypeptides were analyzed through Western blotting. **d** Puromycin-labeled protein levels were quantified and normalized to those of WT hippocampal neurons. *P < 0.05, paired *t* test from 3 WT and KO pairs. **e**, **f** Dock4 cDNA was transfected into Neuro-2a cells, which were then treated with the Rac1 inhibitor NSC23766 (50 μM). The levels of puromycin-labeled proteins were examined (**e**) and were quantified and normalized to the levels in the vector group (**f**). **P < 0.01, ns, no significant, one-way ANOVA with Bonferroni’s Multiple Comparison Test from 3 independent experiments. **g**, **h** cDNAs encoding full-length (FL) Dock4 and its truncation mutants were transfected into Neuro-2a cells. Puromycin-labeled proteins were examined (**g**) and were quantified and normalized to the levels in the vector group (**h**). *P < 0.05, **P < 0.01, ***P < 0.001, ns, no significant, one-way ANOVA with Bonferroni’s Multiple Comparison Test from 3 independent experiments. **i**, **j** Rac1-WT and Rac1-CA (G12V) cDNAs were transfected into Neuro-2a cells. Puromycin-labeled proteins were examined (**i**) and were quantified and normalized to the vector group (**j**). *P < 0.05, **P < 0.01, one-way ANOVA with Bonferroni’s Multiple Comparison Test from 3 independent experiments. **k**, **l** Rac1-GTP (activated form of Rac1) levels in *Dock4* WT and KO hippocampus were examined (**k**) and quantified (**l**). *n* = 3 independent experiments. *P < 0.05, unpaired *t* test. **m**, **n** Dock4 was knocked down in rat hippocampal neurons and WT-Rac1 was delivered into the Dock4-knockdown neurons by using a lentiviral vector. Puromycin-labeled protein levels were analyzed (**m**) and were quantified and normalized to the level in the Dock4-scr group (**n**). *P < 0.05, ^#^*P* < 0.05, ns, no significant, one-way ANOVA with Bonferroni’s Multiple Comparison Test from 3 independent experiments. **o**, **p** Expression levels of AMPAR and NMDAR subunits were examined (**o**) after Dock4 knockdown plus Rac1 overexpression in hippocampal neurons. AMPAR and NMDAR subunit levels were quantified and normalized to the levels in the Dock4-scr group (**p**). *P < 0.05, **P < 0.01, ^#^*P* < 0.05, ^##^*P* < 0.01, ^###^*P* < 0.001, ns, no significant, one-way ANOVA with Bonferroni’s Multiple Comparison Test from 3 independent experiments. α-tubulin (**a**, **c**, **o**) and GAPDH (**e**, **g**, **i**, **k**, **m**) served as loading controls. Error bars: SEM

The diminished expression of glutamate receptors could be resulted from excessive protein degradation or attenuated mRNA translation. To test these possibilities, we used primary cultured hippocampal neurons. Indeed, shRNA-mediated Dock4 knockdown recapitulated the findings in the KO hippocampus: we again observed reduced protein expression of GluA2, GluN1, GluN2A, and GluN2B (Fig. 5a, b). Notably, treatment with MG132, an inhibitor of proteasome-mediated protein degradation, failed to restore the receptor expression (Fig. 5a, b), which indicates that the reduction in protein levels was potentially caused by abrogated protein synthesis/mRNA translation.

To measure protein synthesis, we used puromycin, an aminonucleoside antibiotic structurally similar to aminoacyl-tRNAs, to label newly synthesized polypeptides [41, 42]. Notably, global protein synthesis was significantly lower in hippocampal neurons cultured from *Dock4* KO mice than WT littermates, as indicated by diminished labeling of puromycin (Fig. 5c, d). Because Dock4 functions as a Rac1 GEF, we tested whether Dock4 affects protein synthesis through Rac1 activation; Dock4 overexpression induced protein synthesis, but this effect was negated by the Rac1 inhibitor NSC23766 (Fig. 5e, f). Accordingly, a Dock4 truncated mutant lacking the entire Rac1-activating domain (945VS) failed to promote protein synthesis (Fig. 5g, h), whereas overexpression of WT-Rac1 or its constitutively active (CA) mutant induced protein synthesis (Fig. 5i, j). Notably, the active (GTP-bound) form of Rac1 was drastically decreased in the KO hippocampus (Fig. 5k, l), which suggests that Rac1 activation is diminished when Dock4 is deficient. To verify whether Dock4-Rac1 signaling is a crucial mechanism underlying the protein synthesis of AMPAR and NMDAR subunits, we replenished activated Rac1 in *Dock4*-deficient neurons by lentivirus-mediated delivery of WT-Rac1. Intriguingly, Rac1 overexpression restored global protein synthesis to normal levels in *Dock4*-deficient neurons (Fig. 5m, n), and more importantly, the overexpression reversed Dock4 knockdown-mediated reduction of GluA2, GluN1, GluN2A, and GluN2B (Fig. 5o, p). Collectively, our results revealed that Dock4 is critical for the normal expression of AMPARs and NMDARs, which probably occurs through Rac1-dependent protein synthesis.

### Social behavior is restored in *Dock4* KO mice by increasing Rac1 activity or NMDAR function

**Fig. 6:**
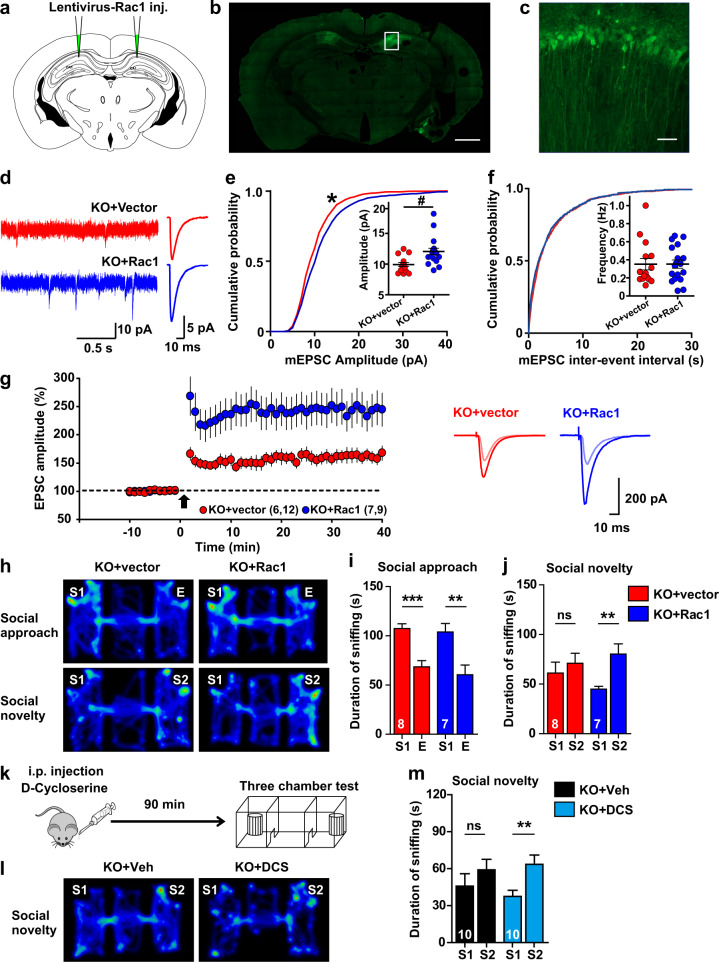
Impaired social behavior of *Dock4* KO mice is rescued by enhancing Rac1 activity or NMDAR function. **a** Rac1-lentivirus was bilaterally injected into the hippocampal CA1 region of *Dock4* KO mice. **b** GFP signals observed in CA1 pyramidal neurons at 4 weeks post-injection confirmed successful Rac1 or control vector expression. Scale bar, 1000 μm. **c** Higher-magnification view of boxed CA1 area in **b**. Scale bar, 40 μm. **d** Representative (left) and average (right) traces of mEPSCs recorded from KO pyramidal cells expressing either vector (KO + vector) or Rac1 (KO + Rac1). **e**, **f** Cumulative probability of mEPSC amplitude (**e**) and inter-event interval (**f**). Insets: average mEPSC amplitude (**e**) and frequency (**f**). *n* = 15 cells from 4 KO + vector mice; *n* = 18 cells from 4 KO + Rac1 mice. *P < 0.05, two-sample Kolmogorov–Smirnov test. ^#^*P* < 0.05, unpaired *t* test. **f** mEPSC frequency from KO + vector or KO + Rac1 cells. **g** Time-course changes of EPSC amplitude (% of baseline) after a pairing protocol (0 mV, 2 Hz, 360 pulses; arrow), measured from KO + vector (12 cells, 6 mice) or KO + Rac1 (9 cells, 7 mice) groups. *P* = 0.021, unpaired *t* test. Representative traces before and at 25–35 min after pairing are shown on the right. **h** Representative heatmap of movement of KO + vector or KO + Rac1 mice in the Three-chamber test at 4 weeks after virus injection. **i**, **j** Duration spent by KO + vector or KO + Rac1 mice in sniffing different cups during social approach (**i**) and social novelty (**j**) phases. E, empty cup; S1, cup containing stranger mouse #1; S2, cup containing stranger mouse #2. *n* = 8 KO + vector mice (4 males and 4 females), and *n* = 7 KO + Rac1 mice (4 males and 3 females). **P < 0.01, ***P < 0.001, ns, no significant, unpaired *t* test. **k** Outline of experimental design for D-cycloserine (DCS) treatment. **l** Representative heatmap of movement of KO mice in social novelty phase at 90 min after administration of DCS (20 mg/kg; KO + DCS) or vehicle (KO + Veh). **m** Duration spent by KO + Veh or KO + DCS mice in sniffing different cups during social novelty phase. *n* = 10 KO + Veh mice (5 males and 5 females), and *n* = 10 KO + DCS mice (5 males and 5 females). *P < 0.05, unpaired *t* test. *n* values of each group are displayed on the corresponding bars of the bar charts. Error bars: SEM

To test whether Rac1 overexpression rescues the behavioral deficits in *Dock4* KO mice, lentiviral particles expressing WT-Rac1 or control vector were bilaterally injected into the hippocampal CA1 region of *Dock4* KO mice (Fig. 6a). A large population of CA1 pyramidal neurons were successfully infected 4 weeks after injection, and the surgery did not affect the locomotion of the animals (Fig. 6a–c, Supplementary Fig. 13a). Notably, synaptic transmission properties, including mEPSC and LTP, were largely rescued in Rac1-expressing KO neurons (Fig. 6d–g). We next measured whether social deficits in *Dock4* KO mice were rescued by Rac1. Intriguingly, Rac1-injected mice showed normal social novelty preference, whereas vector-injected KO mice failed to distinguish unfamiliar from familiar conspecifics (Fig. 6h–j, Supplementary Fig. 13b, c). Thus, replenishing Rac1 in the hippocampal CA1 region was sufficient for correcting defective social preference caused by *Dock4* deficiency.

As described earlier, in *Dock4* KO mice, the NMDAR/AMPAR ratio and NMDAR-dependent LTP were decreased, and concomitantly, NMDAR subunit expression was diminished (Fig. 4c, e, h, i). Therefore, NMDAR hypofunction might represent a major functional cause for the behavioral deficits in the KO mice. To test this hypothesis, we used D-cycloserine (DCS), a partial agonist of NMDAR that holds potential for use in treating autism-like behaviors [43, 44]. Notably, intraperitoneal injection (i.p.) of DCS rapidly restored normal social novelty preference of KO mice (Fig. 6k–m, Supplementary Fig. 13e), but the effect was diminished by 7 days after treatment (Supplementary Fig. 13d, f, g). We also tested the effect of potentiating AMPAR function by PF-4778574, a positive allosteric modulator of AMPAR previously shown to restore social behaviors in ASD mouse models [45]. However, i.p. of PF-4778574 had no effect on correcting social deficit in *Dock4* KO mice (Supplementary Fig. 13h–j). These results suggest that Dock4-Rac1 signaling-mediated NMDAR function, at least in the hippocampal CA1 region, is essential for social novelty preference.

## Discussion

Associations of *DOCK4* variants with ASD have been reported previously, but how this contributes to ASD pathology has remained unexplored. The present study provides novel evidence indicating that *Dock4* deficiency in mice causes ASD-like behaviors, including social and vocalization deficits, elevated anxiety levels, and cognitive dysfunction. Examination of the *Dock4* KO hippocampus revealed abnormalities at excitatory synapses, such as decreased dendritic spine density, impaired AMPAR and NMDAR functions, and abrogated LTP. Notably, we revealed for the first time that normal protein synthesis of GluA2 and three major NMDAR subunits, GluN1, GluN2A, and GluN2B, was regulated by Dock4 through a Rac1-dependent mechanism. Rac1 overexpression in *Dock4*-deficient hippocampal CA1 region was sufficient for correcting social behavior, and pharmacological activation of NMDAR function (by using the NMDAR partial agonist DCS) completely restored social novelty preference in *Dock4* KO mice. Together, the findings of this study identify Dock4-Rac1-dependent regulation of NMDAR function in hippocampus as a previously unrecognized mechanism controlling social behavior.

Both upregulation and downregulation of the synthesis of synaptic proteins have been associated with ASD-like behaviors [46]. Elevated protein synthesis is a major cause of aberrant synaptic transmission and behavioral abnormalities in fragile X syndrome, a disease highly comorbid with ASD [47], but insufficient protein synthesis also leads to ASD-like behaviors. For instance, interference with basal protein synthesis was found to alter spine structure and repetitive behaviors in normal mice [48]. Moreover, several mouse models exhibit ASD features accompanied by reduced translation, and treatment with a translation-boosting small molecule, ISRIB, can reverse social deficits in one of these models [49–51]. Here, we have revealed that social novelty preference requires normal protein synthesis of glutamate receptors, which depends on Dock4 and its target Rac1. Protein synthesis in multiple brain regions has been shown to be necessary for the establishment of social memory, and the hippocampus plays a central role in the integration of these brain regions for social memory storage and consolidation [52–56]. Our findings further show that protein synthesis of glutamate receptors, particularly NMDARs, in the hippocampal CA1 region is crucial for social novelty preference.

Rac1 is a well-studied actin regulator required for normal synaptic function that acts through diverse downstream effectors. Our findings identify a previously unknown role of Rac1, activated by Dock4, in promoting neuronal protein synthesis. Thus, Rac1, as a converging factor that receiving signals from different ASD genes, might exert dual effects on actin dynamics as well as protein synthesis depending on the context of synaptic regulation. The fact that normalization of Rac1 activity corrects ASD-like behaviors in a variety of animal models suggests that targeting Rac1 may be a potential therapeutic approach for ASD. There could be several mechanisms through which Rac1 regulates protein synthesis. First, Rac1 appears to activate mTOR and its downstream target p70S6K (70-kDa ribosomal S6 kinase) [57, 58], a kinase essential for switching on translation initiation [48, 49]. Moreover, active Rac1 recruits CYFIP (cytoplasmic FMRP1-interacting protein) from the CYFIP-FMRP1 complex [59], and this Rac1-dependent shuttling of CYFIP interferes with the translation-inhibition function of FMRP1 and thereby leads to translational activation. Further investigation is required to delineate the mechanism of Dock4/Rac1-dependent protein synthesis under normal and ASD conditions.

Bidirectional changes of E/I ratio of synaptic inputs in different brain regions have been implicated in ASD pathology [60]. The present study reports reduced excitatory and intact inhibitory synaptic transmission in CA1 pyramidal cells of *Dock4* KO mice, leading to an overall inhibition of these cells. Our findings further unravel that NMDAR hypofunction-induced failure of excitatory synaptic function is a main cause of social deficit in *Dock4* KO mice, as NMDAR-mediated synaptic transmission and plasticity were impaired, and the NMDA agonist DCS restored normal social behavior. These results support the view that normalization of NMDAR function, which show effects for amelioration of core symptoms in a number of ASD animal models, is a promising therapeutic strategy for the disease [61]. Moreover, GluN2B-mediated neurotransmission was weakened in the *Dock4* KO hippocampus, which suggests that the GluN2B/GluN2A ratio was lowered at the synaptic membrane. Variations in the gene encoding GluN2B have been frequently identified in neuropsychiatric disorders [62, 63], and studies on animal models have shown that GluN2B is particularly critical for social behaviors [61, 64, 65]. The findings obtained in the present study provide further evidence indicating a crucial role of GluN2B-dependent NMDAR function in ASD pathology.

## Supplementary information

Supplementary Information

Supplementary Table 1

Supplementary Table 2

Supplementary Table 3

Supplementary Table 4

Supplementary Video 1

Supplementary Video 2

Supplementary Video 3
