# Supplementary material for: Autism-like social deficit generated by Dock4 deficiency is rescued by restoration of Rac1 activity and NMDA receptor function
Source: Mol Psychiatry. 2019 Aug 6;26(5):1505–19. doi: 10.1038/s41380-019-0472-7 (PMC8159750; doi:10.1038/s41380-019-0472-7)
Supplement: Supplementary file 1 — Supplementary Information [file 41380_2019_472_MOESM1_ESM.docx]

**Autism-like social deficit generated by *Dock4* deficiency is rescued by restoration of Rac1 activity and NMDA receptor function**

Daji Guo, Yinghui Peng, Laijian Wang, Xiaoyu Sun, Xiaojun Wang, Chunmei Liang, Xiaoman Yang, Shengnan Li, Junyu Xu, Wen-Cai Ye, Bin Jiang, Lei Shi

**Supplementary Figure**


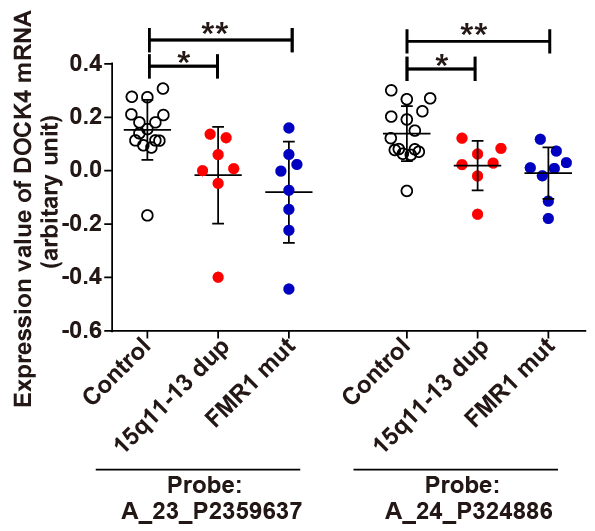


**Supplementary Figure 1 Evidence of *DOCK4* mRNA change in autism spectrum disorder (ASD).** Data from Gene Expression Omnibus (GEO) profile database show that *DOCK4* mRNA was significantly decreased in lymphoblastoid cells of two populations of ASD patients, those with fragile X mutation (FMR1-mut) or with a 15q11–q13 duplication (15q11-13 dup) respectively [^1^](#_ENREF_1).

**
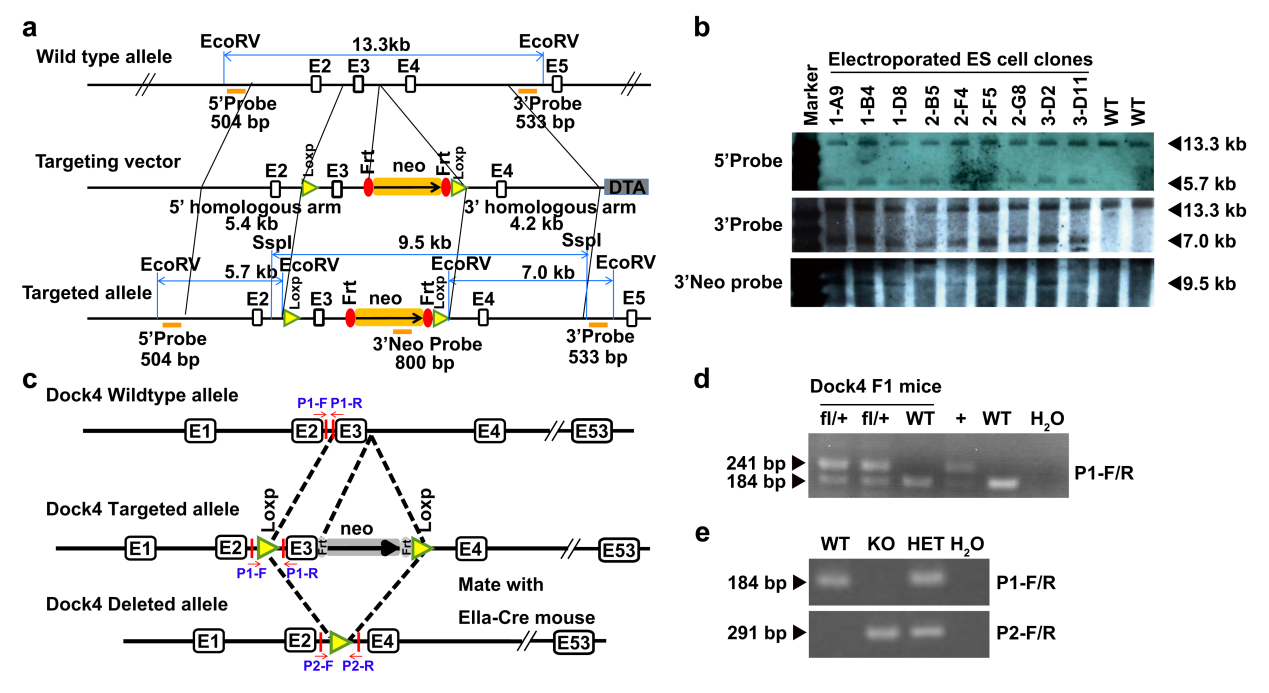
**

**Supplementary Figure 2 Generation of *Dock4* KO mice.** (**a**) Strategy for homologous recombination of *Dock4* gene in ES cells. The targeting construct contains a 5.4 kb 5’ homologous arm and a 4.2 kb 3’ homologous arm fragments. E, exon. DTA, diphtheria toxin A. Locations of the probes for Southern blotting are indicated. The EcoRV and SspI digestion sites and excised size of *Dock4* DNA fragments in wild type and mutant alleles are also indicated. (**b**) Result of Southern blot analysis with 5’probe, 3’probe and 3’Neo probe. Genomic DNAs from 9 electroporated ES cell clones were digested with EcoRV and SspI, and hybridized with the probe. Eight positive cell clones were identified (1-A9, 1-D8, 1-B4, 2-F4, 2-F5, 2-G8, 3-D2, 3-D11). WT, wild type. (**c**) Strategy used for generating *Dock4* KO mice. Locations of Primers for genotyping are indicated. P1-F/R, forward/reverse primer 1; P2-F/R, forward/reverse primer 2. (**d**) Representative genotyping result of F1 mice by PCR analysis. Germline transmission of the mutant allele could be confirmed by the presence of both 184 bp and 241 bp PCR products using P1-F/R. (**e**) Genotypes of *Dock4* WT, HET, and KO mice were determined by PCR using both P1-F/R and P2-F/R.

**
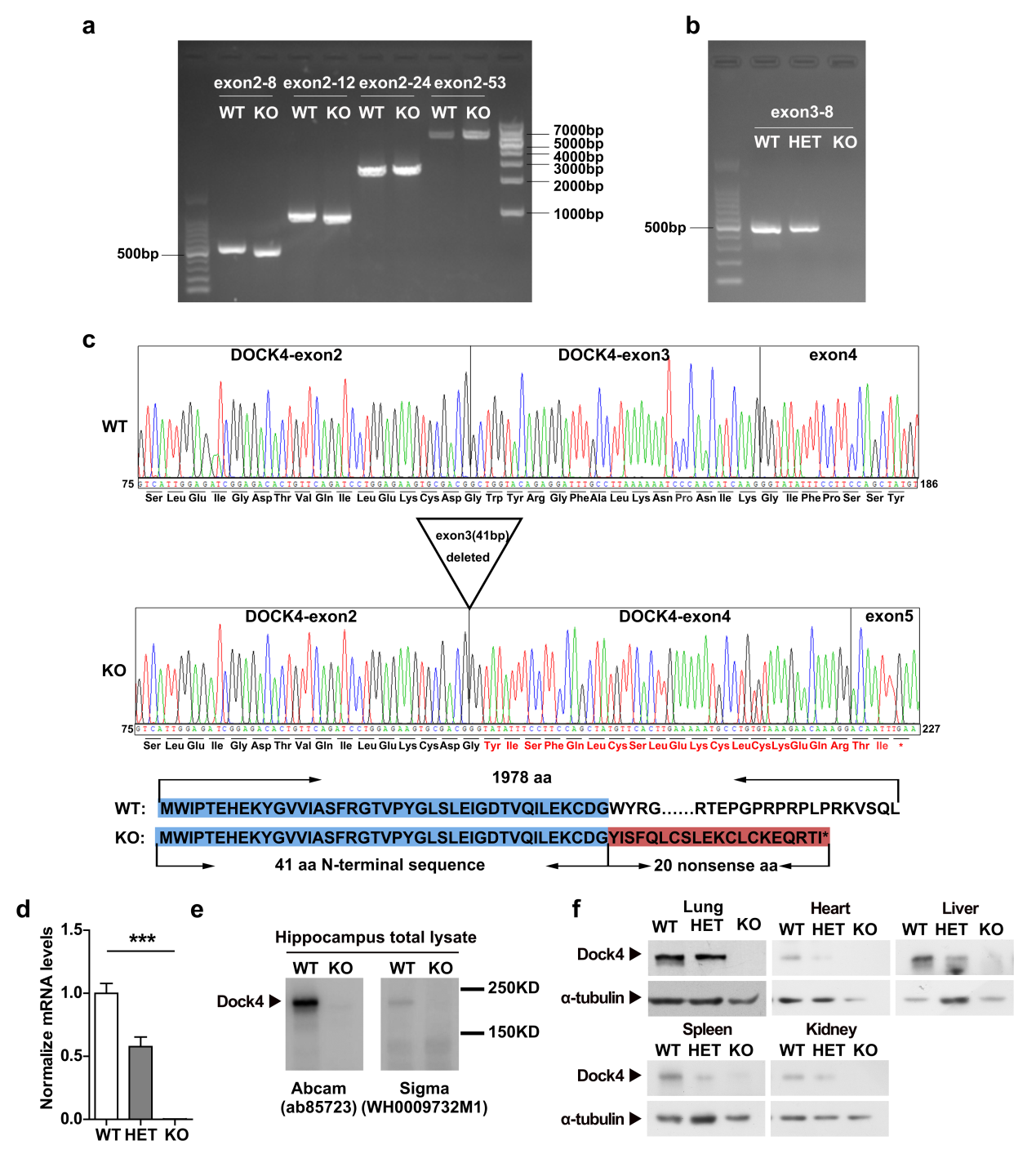
**

**Supplementary Figure 3 Validation of *Dock4* KO mice.** (**a**) Different sizes of PCR products of *Dock4* mRNA from WT and KO brain using forward primer in Exon 2 and different reverse primers in Exon 8, 12, 24 and 53 respectively. (**b**) No PCR product was detected in KO mice using forward primer in Exon 3 and reverse in Exon 8. (**c**) Sequencing results from WT and KO PCR products confirmed that a transcript without Exon 3 (41 bp) replaced the full-length *Dock4* transcript in KO mice, leading to coding frame shift after Exon 2 with premature stop. The truncated protein contains 41 aa N-terminal sequence of Dock4 followed by 20 nonsense aa due to frameshift. (**d**) *Dock4* mRNA levels were assessed by quantitative RT-PCR, and were normalized to those of WT mice. Values represent mean ± SEM from 6 WT, HET or KO mice. ****P*<0.001, one-way ANOVA with Bonferroni’s Multiple Comparison Test. (**e**) Protein expression analysis of Dock4 in hippocampus using two Dock4 antibodies, suggesting complete deletion of Dock4 full length protein in KO mice. (**f**) Protein expression analysis of Dock4 in lung, heart, liver, spleen and kidney from WT, HET, and KO mice. α-tubulin served as a loading control.

**
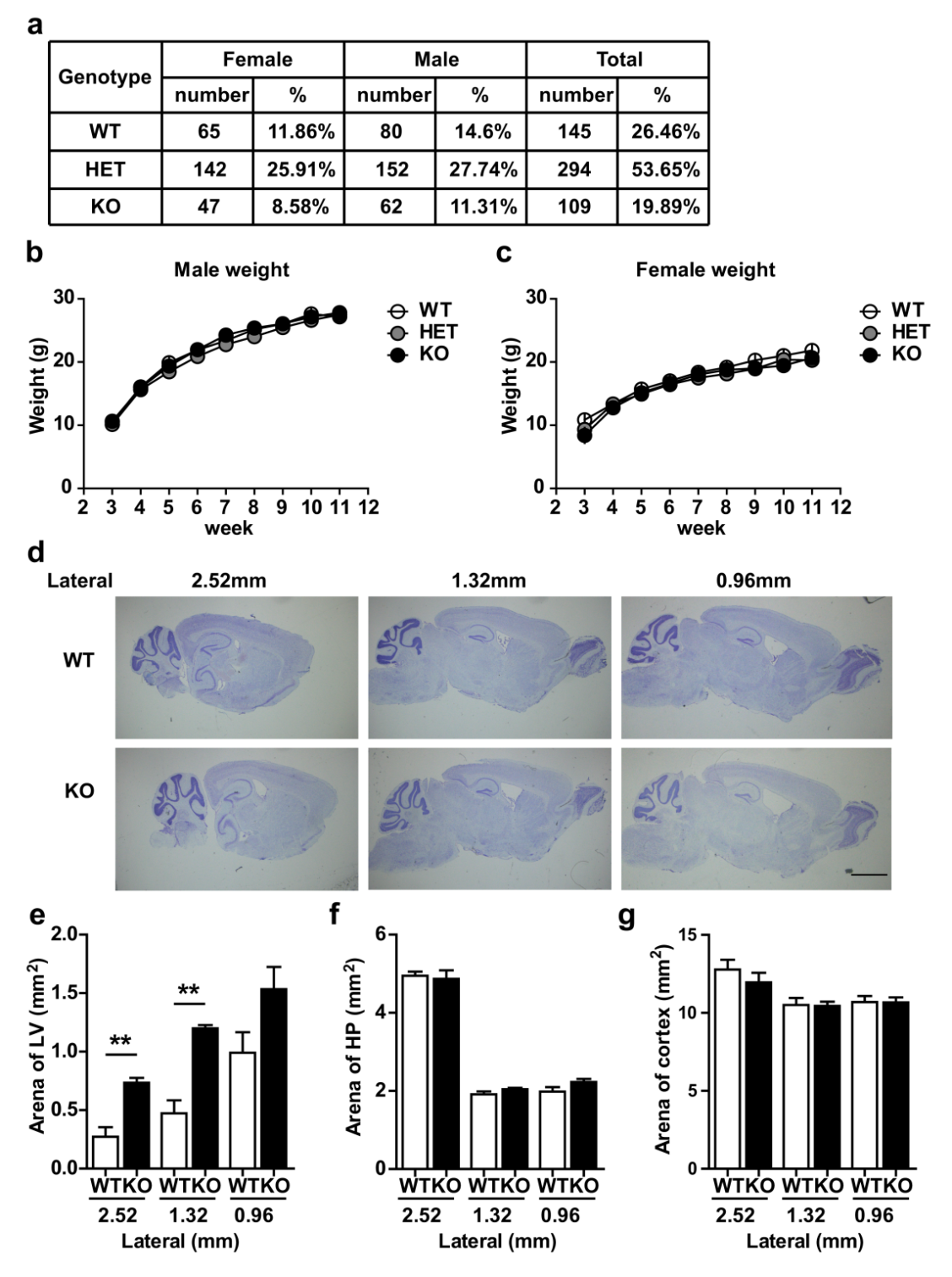
**

**Supplementary Figure 4 Characterization of Dock4 KO mice.** (**a**) The birth rate of *Dock4* WT, HET, and KO mice was calculated. (**b,c**) The body weight of male (b) and female (c) HET and KO mice was similar compared to their WT littermate from postnatal week 3 to 11. Values represent mean ±SEM. n=28 WT males and 25 WT females, n=61 HET males and 41 HET females, and n=22 KO males and 19 KO females; one-way ANOVA with Bonferroni’s Multiple Comparison Test. (**d**) Nissl staining of sagittal brain sections from 5-month-old WT and KO mice. Scale bar, 2 mm. (**e-g**) Quantification of the areas of lateral ventricle (e), hippocampus (f) and cortex (g) from sagittal brain sections at different locations (lateral 2.52mm, 1.32mm, 0.96mm respectively). Values represent mean ± SEM from 3 WT or KO mice. ***P*<0.01, unpaired *t* test.

**
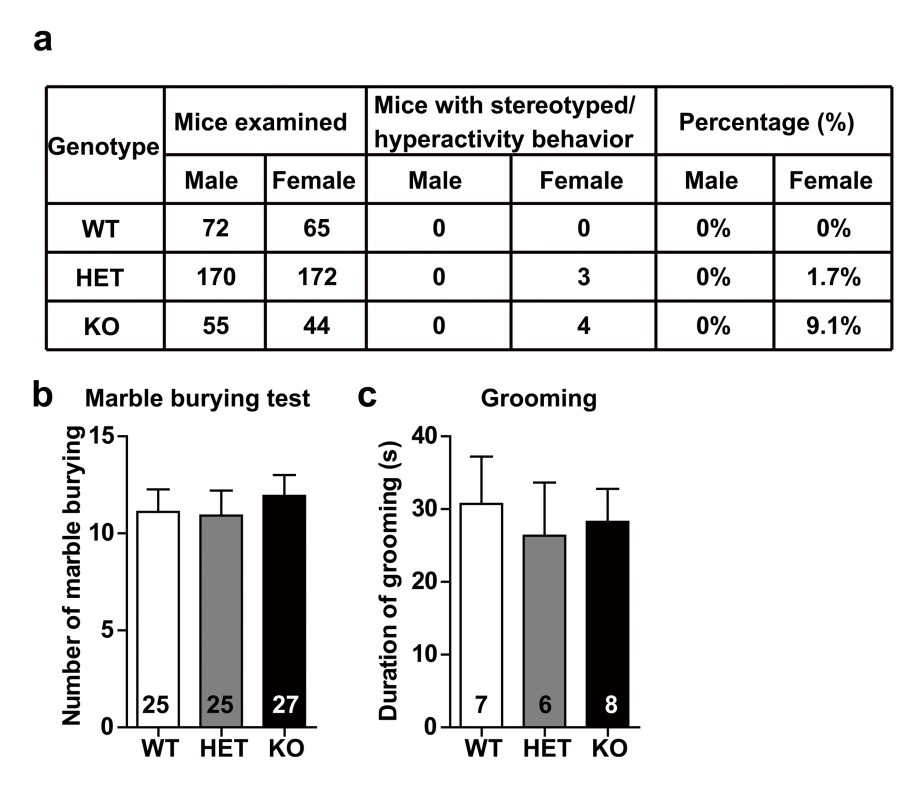
**

**Supplemental Figure 5 A small population of *Dock4* KO mice exhibit repetitive and stereotyped patterns of behavior. (a)** 1.7% female *Dock4* HET female mice and 9.1% female KO mice exhibited autism-like stereotyped behavior such as continuous circling (also see Supplementary Video 1–3). (**b**) Mice that did not show stereotyped circling were subjected to marble burying test. The numbers of marbles buried by *Dock4* WT, HET, and KO mice were similar. n=25 WT mice (17 males and 8 females), n=25 HET mice (17 males and 8 females) and n=27 KO mice (16 males and 11 females); One-way ANOVA with Bonferroni's Multiple Comparison Test. (**c**) Time spent by mice of different genotypes for grooming was measured in a 10 min session. n=7 WT male mice, n=6 HET male mice and n=8 KO male mice; One-way ANOVA with Bonferroni’s Multiple Comparison Test. Error bars: SEM.

**
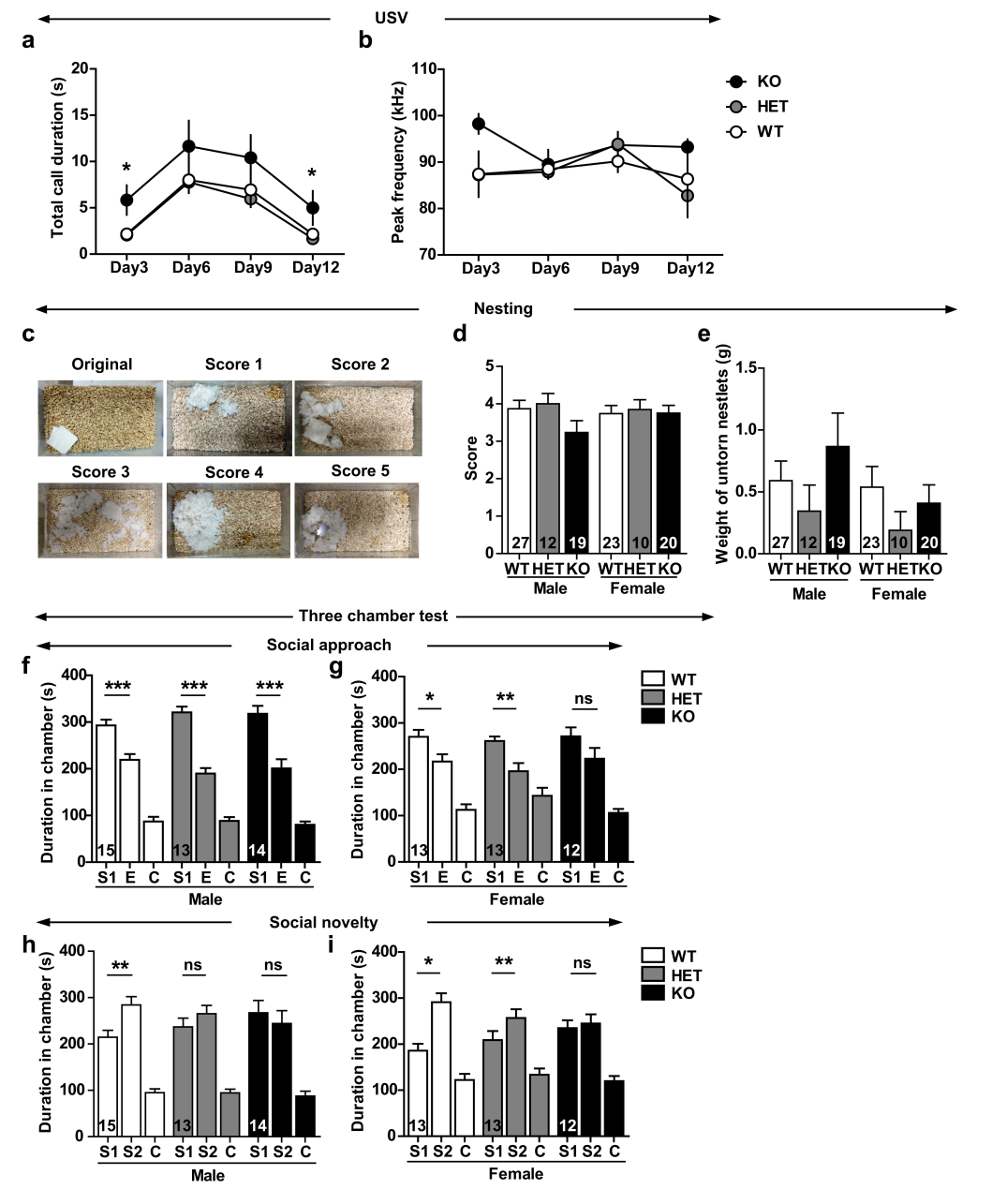
**

**Supplemental Figure 6 *Dock4* KO mice exhibit vocal and social deficits.** (**a,b**) Maternal separation-induced pup ultrasonic vocalizations were measured at postnatal day (P) 3, P6, P9, and P12. Total call duration (a) and peak frequency (b) were analyzed. n = 28, 28, 29, and 26, respectively for WT; n = 39, 41, 38, and 38, respectively for HET; n = 13, 15, 15, and 13, respectively for KO. *P<0.05, one-way ANOVA with Bonferroni’s Multiple Comparison Test. (**c**) Nesting behavior of WT, HET, and KO mice was assessed after overnight introduction of Nestlets. Representative nests built by different genotypes are shown. (**d**) The nest qualities were scored and analyzed using Kruskal-Wallis test with Dunn’s Multiple Comparison Test. (**e**) Weight of untorn Nestlets after introduction overnight. One-way ANOVA with Bonferroni’s Multiple Comparison Test. (**f–i**) Duration spent in different chambers by male (f,h) and female (g,i) WT, HET and KO mice in social approach (f,g) and social novelty (h,i) phases; E, chamber with an empty cup; S1, chamber with a cup containing stranger mouse #1; S2, chamber with a cup containing stranger mouse #2; C, the center chamber. **P*<0.05, ***P*<0.01, ****P*<0.001, ns, no significant, one-way ANOVA with Bonferroni’s Multiple Comparison Test. n values of each group are displayed on the corresponding bars of the bar charts. Error bars: SEM.

**
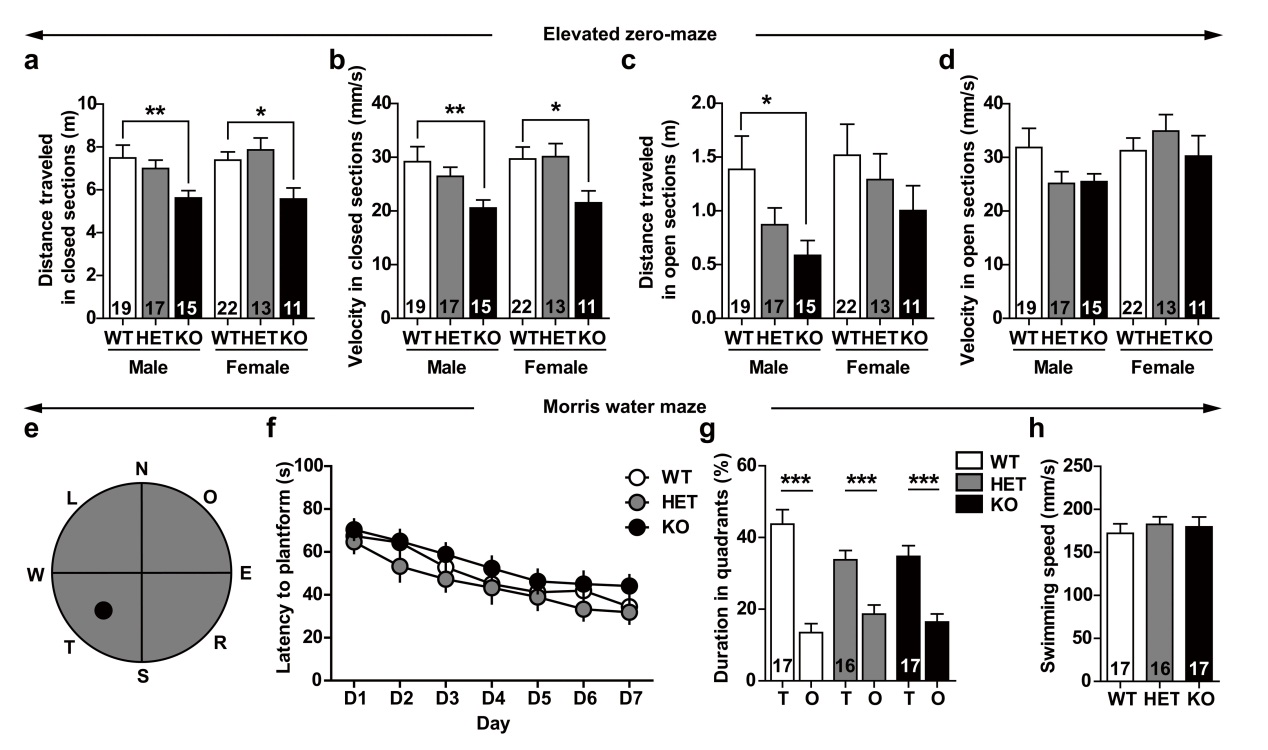
**

**Supplemental Figure 7 *Dock4* KO mice exhibit increased anxiety levels in elevated zero-maze and normal learning in Morris water maze.** (**a-d**) *Dock4* WT, HET and KO mice were tested in elevated zero-maze, and the following variables were measured and analyzed: travel distance (**a**) and velocity (**b**) in closed sections; travel distance (**c**) and velocity (**d**) in open sections. n values of each group are displayed on the corresponding bars of the bar charts. **P*<0.05, ***P*<0.01, one-way ANOVA with Bonferroni’s Multiple Comparison Test. (**e**) Designated quadrants in Morris water maze. (**f**) Latency in locating the submerged platform was measured during 7-day training of Morris water maze test. (**g**) Duration that the mice spent in the target or opposite quadrant 24 h after the last trial of training. (**h**) Swim speed of WT, HET and KO mice was similar. n=17 WT mice (7 males and 10 females), n=16 HET mice (7 males and 9 females) and n=17 KO mice (9 males and 8 females). ****P*<0.001, unpaired *t* test. Error bars: SEM.


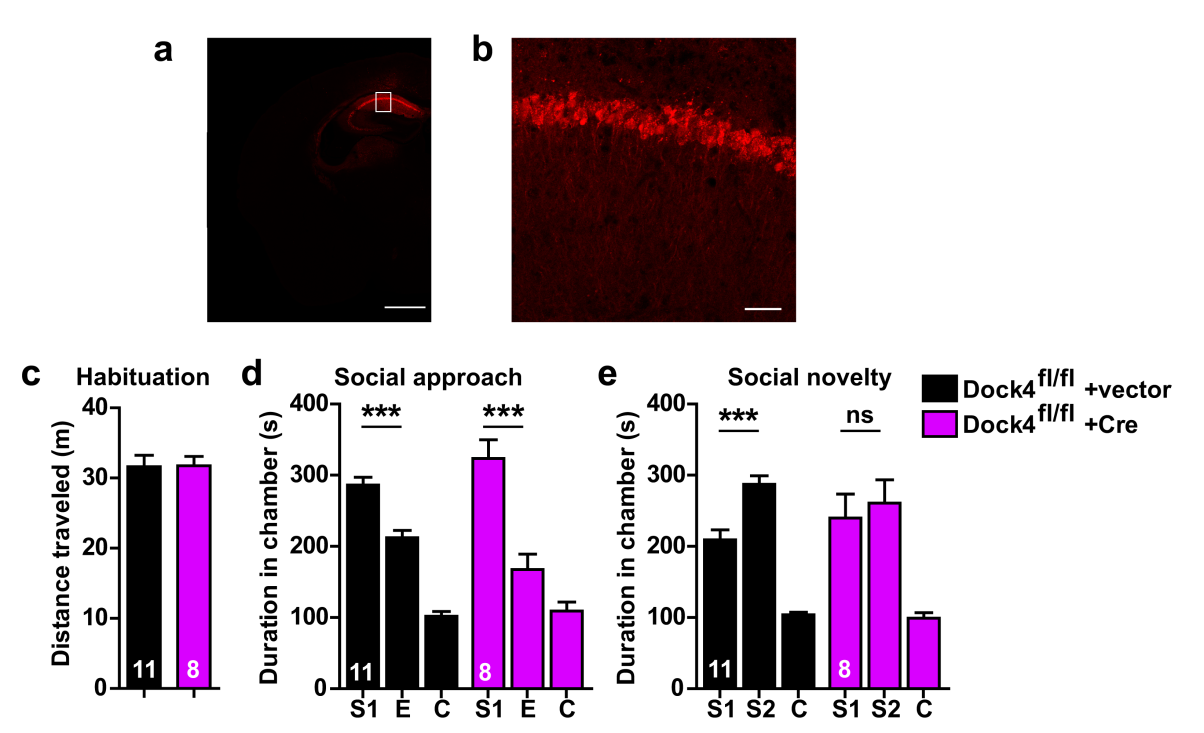


**Supplementary Figure 8 Deletion of *Dock4* in hippocampus leads to impaired social novelty preference.** (**a**) mCherry signals observed in CA1 pyramidal neurons at four weeks post-injection confirmed successful Cre expression. Scale bar, 1000 μm. (**b**) Higher-magnification view of boxed CA1 area in (a). Scale bar, 40 μm. (**c**) Distanced traveled by mice with hippocampal deletion of *Dock4* and control mice during habituation phase. (**d-e**) Duration spent in different chambers by the two groups of mice in social approach (d) and social novelty (e) phases; E, chamber with an empty cup; S1, chamber with a cup containing stranger mouse #1; S2, chamber with a cup containing stranger mouse #2; C, the center chamber. ****P*<0.001, ns, no significant, one-way ANOVA with Bonferroni’s Multiple Comparison Test. n=11 *Dock4^fl/fl^* + AAV-vector mice (6 males and 5 females), and n=8 *Dock4^fl/fl^* + AAV-Cre mice (4 males and 4 females). Error bars: SEM.


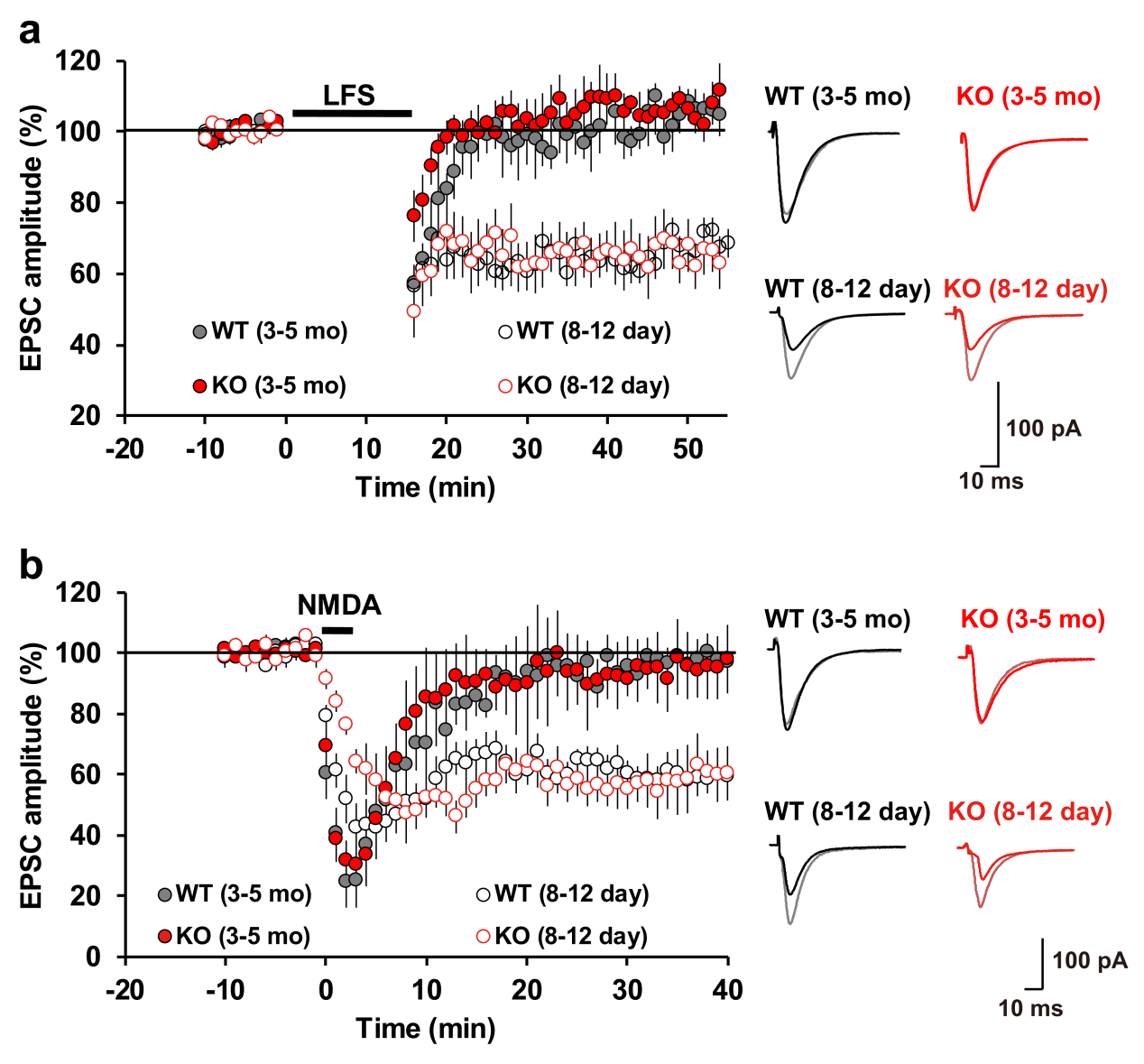


**Supplementary Figure 9 LTD was not impaired in *Dock4* KO hippocampus.** (**a**) Time-course changes of EPSC amplitude recorded in CA1 pyramidal cells after LFS at 1 Hz for 15 min (900 pulses) paired with continuous postsynaptic depolarization at -40 mV from WT and KO mice at the age of 8–12 days (WT: 13 cells, 5 mice, KO: 10 cells, 4 mice, of baseline measured 25–35 min after LFS, unpaired *t* test, p=0.76) and the age of 3–5 months (WT: 11 cells, 5 mice, KO: 9 cells, 4 mice, unpaired *t* test, p=0.62). The representative traces before and at 25–35 min after pairing are shown on the right. (**b**) Time-course changes of EPSC amplitude recorded in CA1 pyramidal cells after application of NMDA (10 μM, 3 min) from WT and KO mice at the age of 8–12 days (WT: 12 cells, 5 mice, KO: 8 cells, 4 mice, of baseline measured 25-35 min after the cessation of NMDA application, unpaired *t* test, p=0.71) and the age of 3–5 months (WT: 9 cells, 4 mice, KO: 9 cells, 4 mice, unpaired *t* test, p=0.83). The representative traces before and at 25–35 min after cessation of NMDA application are shown on the right.

**
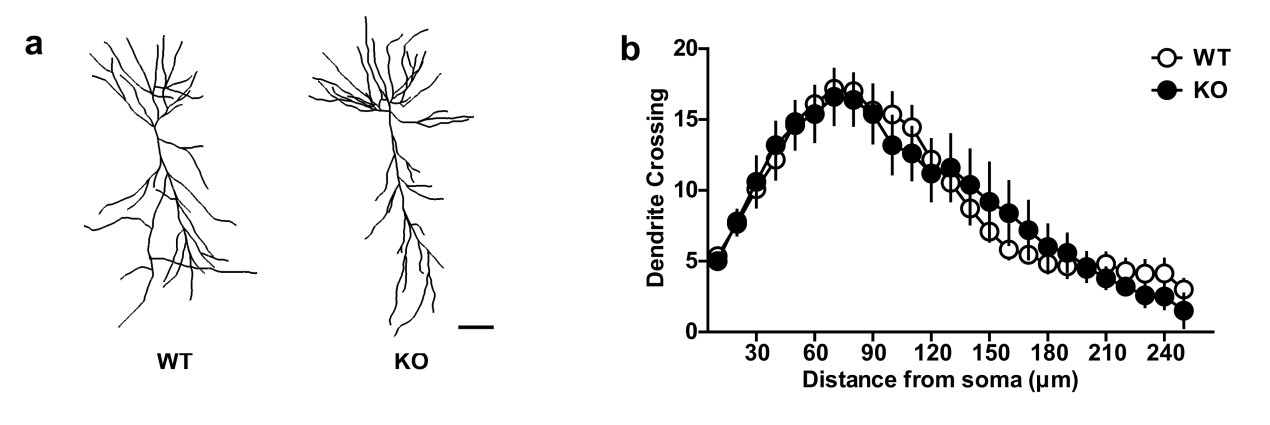
**

**Supplementary Figure 10 Normal dendrite arborization in KO mice hippocampus.** (**a**) Reconstructions of representative Golgi-stained pyramidal neurons from hippocampal CA1 region of WT and *Dock4* KO mice. Scale bar, 50 μm. (**b**) Number of intersections of the dendrites at different distances (radius) from the soma (center of analysis), quantified by Sholl analysis, in WT (n= 11 neurons from 3 mice) and KO (n= 5 neurons from 3 mice). Unpaired *t* test. Error bars: SEM.

**
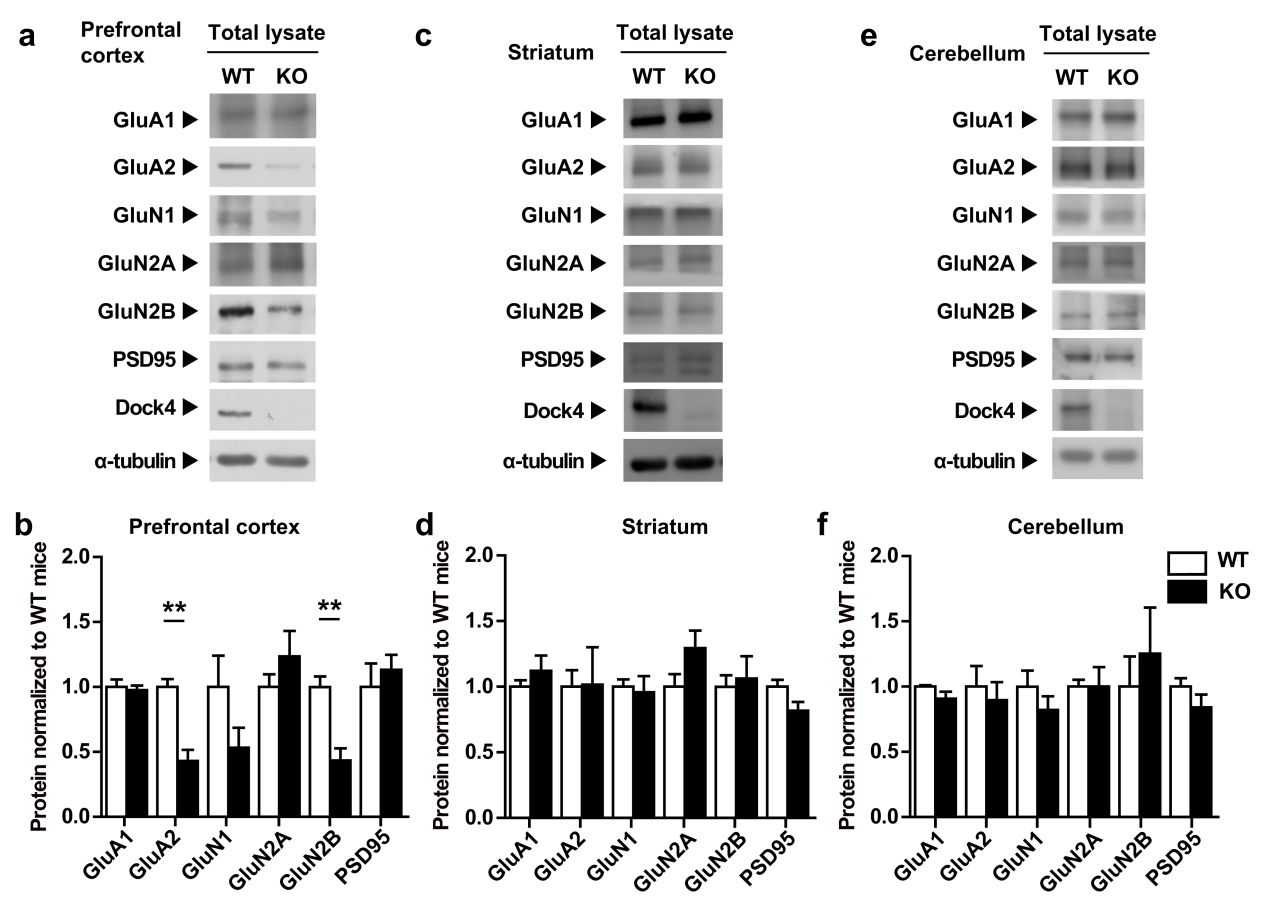
**

**Supplementary Figure 11 Expression of several AMPA and NMDA subunits was decreased in prefrontal cortex, but not striatum and cerebellum.** (**a-f**) Expression levels of AMPAR and NMDAR subunits in total lysate of prefrontal cortex (a), striatum (c) and cerebellum (e) of WT and KO mice. α-tubulin served as a loading control. Quantification of these proteins in prefrontal cortex (b), striatum (d) and cerebellum (f) of WT and KO mice. n=4 WT and KO pairs, ***P*<0.01, unpaired *t* test. Error bars: SEM.

**
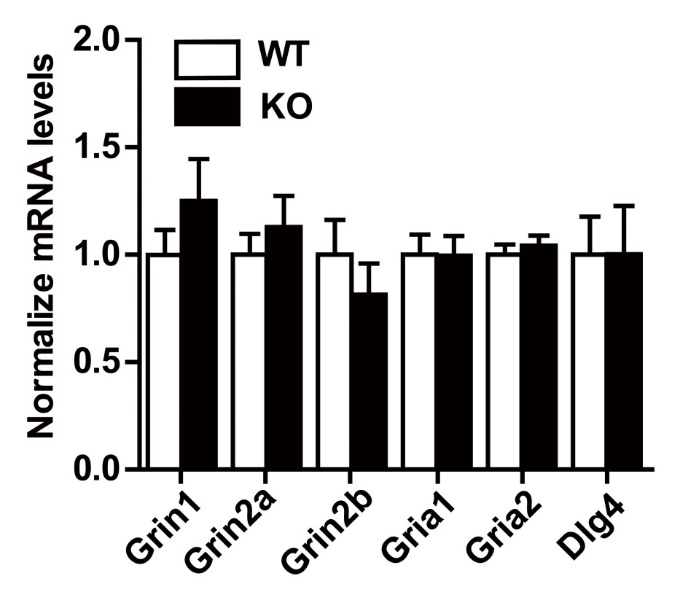
**

**Supplementary Figure 12 mRNA levels of AMPAR and NMDAR subunits and PSD-95 are unaltered in *Dock4* KO mice.** Levels of indicated mRNA in the WT or KO hippocampus were assessed by quantitative RT-PCR. All mRNA levels in KO hippocampus were normalized to those of WT group. n=6 WT and KO mice pairs, unpaired *t* test.

**
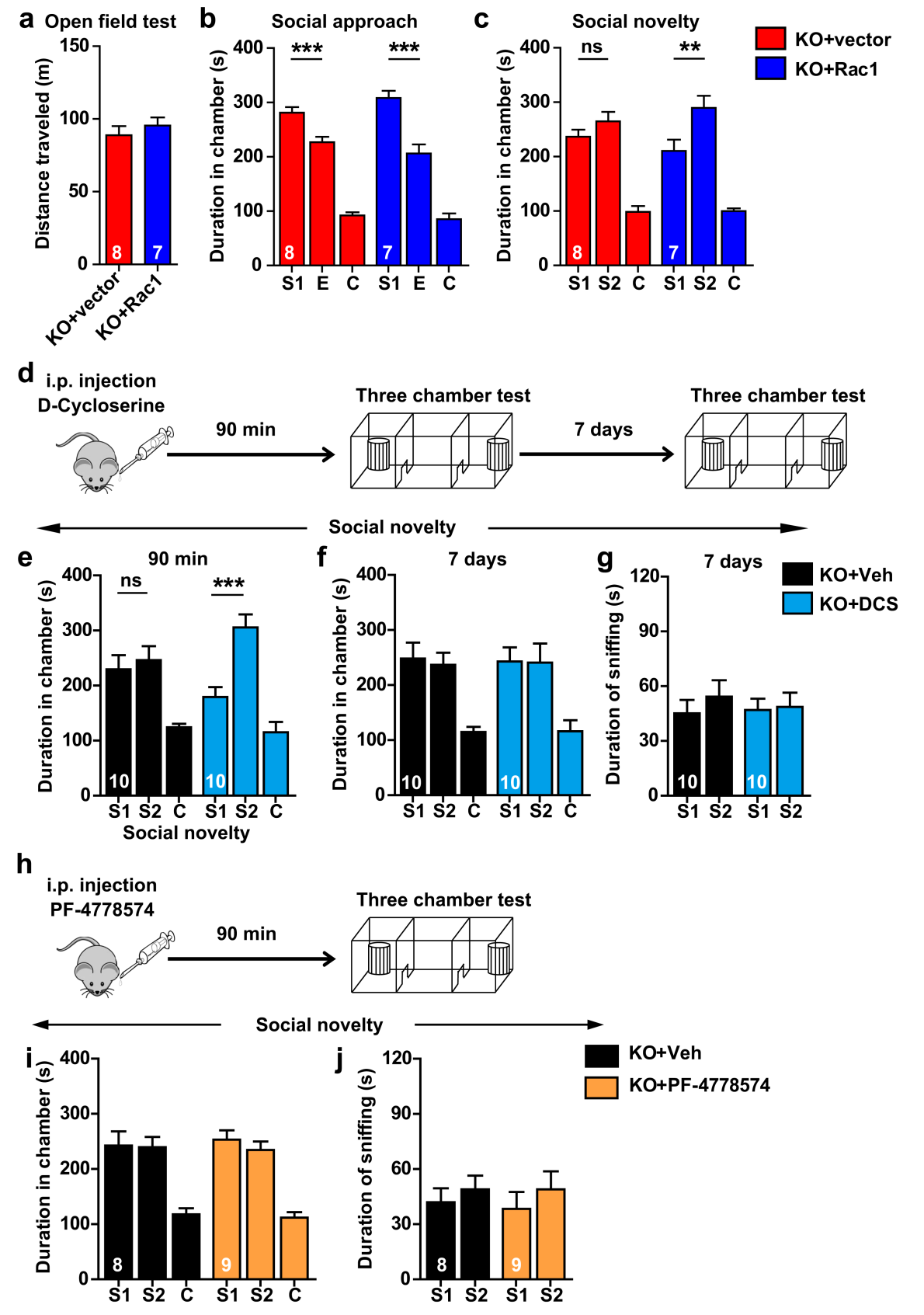
**

**Supplemental Figure 13 Social preference deficit in *Dock4* KO mice is rescued by Rac1 overexpression and NMDAR activation, but not by AMPAR activation.** (**a**) Distance traveled by *Dock4* KO mice in open field at 4 weeks after lentivirus injection. Unpaired *t* test. (**b,c**) Duration spent in different chambers by KO mice during social approach (b) and social novelty (c) phase in the Three-chamber test at 4 weeks after lentivirus injection. n=8 KO mice (4 males and 4 females) injected by vector (KO+vector) and n=7 KO mice (4 males and 3 females) injected by Rac1 (KO+Rac1). E, chamber with an empty cup; S1, chamber with a cup containing stranger mouse #1; S2, chamber with a cup containing stranger mouse #2; C, center chamber. ***P*<0.01; ****P*<0.001, ns, no significant, one-way ANOVA with Bonferroni’s Multiple Comparison Test. (**d**) An outline of the experimental design used for D-cycloserine (DCS) treatment. (**e,f**) Duration in different chambers of KO mice during social novelty phase at 90 min (e) or 7 days (f) after DCS (20 mg/kg) treatment. S1, chamber with a cup containing stranger mouse #1; S2, chamber with a cup containing stranger mouse #2; C, center chamber. (**g**) Duration spent in sniffing different cups of KO mice in social novelty phase at 7 days after DCS administration; S1, cup containing stranger mice #1; S2, cup containing stranger mice #2. n=10 KO mice (5 males and 5 females) treated with vehicle (KO+Veh); n=10 KO mice (5 males and 5 females) treated with DCS (KO+DCS). ****P*< 0.001, ns, no significant, one-way ANOVA with Bonferroni’s Multiple Comparison Test or unpaired *t* test. (**h**) An outline of the experimental design used for PF-4778574 treatment. (**i**) Duration in different chambers of KO mice during social novelty phase at 90 min after PF-4778574 (0.3mg/kg) treatment. S1, chamber with a cup containing stranger mouse #1; S2, chamber with a cup containing stranger mouse #2; C, center chamber. (**j**) Duration spent in sniffing different cups of KO mice in social novelty phase at 90 min after PF-4778574 administration; S1, cup containing stranger mice #1; S2, cup containing stranger mice #2. n=8 KO mice (4 males and 4 females) treated with vehicle (KO+Veh); n=9 KO mice (4 males and 5 females) treated with PF-4778574 (KO+PF-4778574). One-way ANOVA with Bonferroni’s Multiple Comparison Test or unpaired *t* test. Error bars: SEM.

**Supplementary Materials and Methods**

**Generation of *Dock4* knockout (KO) mice**

*Dock4* KO mouse model was created by Beijing Biocytogen. In brief, homology regions covering 5.4 kb upstream of Dock4 exon 3 and 4.2 kb downstream of exon 3 were subcloned from a BAC clone (RP23-200K21; Invitrogen) from C57BL/6J mouse genomic BAC library. FRT-flanked Neo resistance positive selection cassette was inserted downstream of exon 3 and two loxP sites were introduced upstream of exon 3 and downstream of exon 3, respectively. A diphtheria toxin A (DTA) cassette (negative selection marker) was used for the elimination of randomly inserted mouse embryonic stem cells. Two loxP cassettes were located upstream and downstream of the exon 3 of *Dock4* gene respectively. After linearization, the targeting vector was transfected into C57BL/6 embryonic stem (ES) cells (Biocytogen) by electroporation. After selection with G418, single colonies were picked and screened by PCR for successful homologous recombination of the targeting vector. Positive clones were then confirmed by Southern blotting with 5’probe, 3’probe and an internal probe (Supplementary Figure 2a, b). The internal probe was used to eliminate any random insertion of the targeting vector. Positive clones were injected into Balb/c blastocysts and implanted into pseudo-pregnant females to generate chimeric mice, which were crossed with C57BL/6 mice to obtain F1 mice carrying the recombined allele containing the floxed *Dock4* allele and Neo selection cassette (*Dock4*^fl-neo/+^). *Dock4*^fl-neo/+^ mice were crossed with Flp mice (C57BL/6 background) to remove the positive selection marker and were then intercrossed for breeding to obtain mice containing the floxed Dock4 allele (*Dock4*^fl/fl^ mice). Also *Dock4*^fl-neo/+^ mice were crossed with EIIa-Cre (C57BL/6 background) mice to obtain *Dock4^+/-^* mice, which were then intercrossed for breeding to obtain *Dock4^-/-^* (KO), *Dock4^+/-^* (heterozygous, HET) and *Dock4^+/+^* (wild type, WT) mice for tests.

**Genotyping**

Genotyping was performed by PCR using genomic DNA extracted from mouse tails. *Dock4*^fl-neo/+^ mice were determined by the presence of 184 bp and 241 bp products using primer 1 pairs. The genotypes of Dock4 F2 mice and the following generations were confirmed by primer 1 and 2 pairs. The HET mice were determined by the presence of a 184 bp product using primer 1 pairs and a 291 bp product using primer 2 pairs. WT mice were determined by a 184 bp product using primer 1 pairs and no product using primer 2 pairs, while KO mice were determined by no product using primer 1 pairs and a 291 bp product using primer 2 pairs. All primer sequences are listed below: (Sequence from 5’ to 3’)

P1-F: TGGGCTTGGTGGCACATAAC

P1-R: GTACAGGAAATAAGGGCTTGGATCA

P2-F: CAGAGTGGCAGTAAATCTGTGTAG

P2-R: CAACGTGACACTAAAACAAACTTGT

**RNA extraction and quantitative real time (RT)-PCR**

After sacrifice, hippocampi were cut out from WT, HET and KO mice (n=6) and homogenized in Trizol (Takara) and subjected to RNA extraction. cDNA was synthesized using M-MLV Reverse Transcriptase Kit (Promega). qRT-PCR was performed by iQTM SYBR Green Supermix (BIO-RAD) according to manufacturer’s instructions. Briefly, 0.5 μl of cDNA was mixed with 8 μl of 2×iQTM SYBR Green Supermix, and 1 μl of forward and reverse primers (5 μM), and was brought to a total volume of 16 μl with RNase-free water. All samples were performed in triplicate. Target amplification was performed in 96-well plates by using LightCycler 480 Real-Time PCR System (Roche). Primer sequences used for qPCR are listed below: (Sequence from 5’ to 3’)

Dock1-F: TCCTTTCTGGAACTCTGCCC

Dock1-R: CTCACCACCAGGTCCAAATCA

Dock2-F: GGAACGCTACCCAAGGATGAG

Dock2-R: CCTCCTCGTGAGCATGGAAC

Dock3-F: ACTGGGGTAATGAGCATTTGG

Dock3-R: CGCATTGTATCTACTTGGGATGT

Dock4-F: ACGGCTGGTACAGAGGATTTG

Dock4-R: GCTGTTTCCACATGGTTCCC

Dock5-F: CACCTGGTGGACAGATCAACA

Dock5-R: CCCATGGATCAGGGTGACAT

Dock6-F: AGGACTGGGTCATTGTACGC

Dock6-R: AGGGGTATCCTGCTCGAAGA

Dock7-F: CCATCTGGAAGCGCCTTTG

Dock7-R: ACGATGATCTCTAGCGTGTCT

Dock8-F: TCCCTACAGCGAAGAGTCAA

Dock8-R: GTTTGACTGGCAGCATCTCG

Dock9-F: ATCTCCAAGGAACCGAAAGGG

Dock9-R: CGCCAGAAGGTAACTGCTTT

Dock10-F: CAAGGGCTGCATCTTTTTGG

Dock10-R: TTCAGCAGCCAGCACAAAAT

Dock11-F: AGAAAAAGGACACAGTCGAGGC

Dock11-R: TCCGGATGCATGCTCCTTTC

Gria1-F: TAGGCTGCCTGAACCTTTGG

Gria1-R: TGTGGCACTCACTGAAGCAT

Gria2-F: CCCATCAGGAAATGACACGTC

Gria2-R: CGCTCATTCCCTTCAAGCAT

Grin1-F: GGTGTCACTCACCCAACCTT

Grin1-R: ACCGGGATTAGGGGTGGTTA

Grin2a-F: AGAACTCCACGCATTGCAGA

Grin2a-R: CATAGAGGTTCCCCATCCGC

Grin2b-F: CTCAGCGACCTGTATGGCAA

Grin2b-R: CCATAGGTGACAGTGTGCGT

Dlg4-F: TGACAACCAAGAAATACCGCT

Dlg4-R: TTCCATTCACCTGCAACTCATA

GAPDH-F: GAAGGTCGGTGTGAACGGAT

GAPDH-R: TTCCCATTCTCGGCCTTGAC

**Amplification of different *Dock4* mRNA fragments by PCR**

Hippocampal cDNA from WT, HET and KO mice were synthesized as described above. Forward primers in Exon 2 or 3 and different reverse primers in Exon 8, 12, 24, or 53 were used to amplify different *Dock4* mRNA fragments by TaKaRa Taq (Takara). The products were identified with 1% agarose gel. All primer sequences are listed below: (Sequence from 5’ to 3’)

Exon2-F: GTCATTGGAGATCGGAGACAC

Exon3-F: CTGGTACAGAGGATTTGCCTTA

Exon8-R: TCTTCCGATGTCGATGTTCC

Exon12-R: CTGCTAATCCTGCATTGGAGC

Exon24-R: GACAGGCAGGAGAATGTACC

Exon53-R: TTATAGCTGAGACACCTTACGGG

**Dock4 expression and total protein analysis**

After sacrifice, different brain regions (cortex, hippocampus, cerebellum, brain stem, olfactory bulb) and other tissues (heart, liver, spleen, lung and kidney) were quickly dissected out from WT, HET and KO mice. Tissue samples were homogenized in cold RIPA lysis buffer (consisting of 1% NP-40, 0.5% sodium deoxycholate, and 0.1% SDS) containing protease and phosphatase inhibitors (Selleck), and lysed for 30 min. Lysates were cleared by centrifugation at 12,000 rpm (15,294 × g) for 30 min. Protein concentration was determined using a standard BSA method, and 30 µg of total protein for each sample was separated by SDS-PAGE followed by Western blot analysis.

**Brain weighing and Nissl staining**

Adult (5-month-old) mice were anesthetized with 1.25% tribromoethanol (Sigma), then intracardially perfused with PBS followed by 4% paraformaldehyde (PFA). Brains were removed and post-fixed for 24 h in 4% PFA at 4⁰C, then dehydrated by 10–30% gradient sucrose in PBS at 4⁰C for several days until dehydrated completely. Brains were briefly dried on paper tissues and weighed. Then brains were processed for OCT embedding, and sagittal brain sections (30 µm) were cut on a Leica cryostat and mounted on adhesion microscope slides (CITOTEST). The slices were degreased using a descending series of ethanol (100%, 95% and 70%), and were then washed using distilled water. Sections were treated with 1% cresyl-violet for 10 minutes and then washed with distilled water. Sections were then dehydrated using an ascending series of ethanol (70%, 95% and 100%), and were placed in dimethylbenzene, and were finally mounted using neutral resin.

**Behavior tests**

For the generation of animals, HET mice were used for breeding. KO, HET or WT mice used for behavior analyses were littermates. All animals were housed in three or four per cage and provided with food and water *ad libitum*. The housing room was maintained at 23 ± 2⁰C on a 12 h dark/light cycle (lights on/off at 8:00/20:00). Sex matched mice (3–6 months) were used for behavior experiments and were individually caged for at least one week before the beginning of the experiments. To avoid carryover effects, tests were performed in an order from the least to the most stressful levels shown as follows: nesting, pup retrieval test, open field test, Three-chamber test or novel object recognition test, marble burying, grooming, Y-maze (spontaneous alternation or spatial recognition), elevated zero-maze, and Morris water maze. Mice were allowed to rest for 2–3 days between different tests. All tests were performed between 10:00 and 18:00. Mice were transferred to the test room 1 h for habituation before each test. The apparatus was cleaned between trials with 75% ethanol and left several minutes to allow ethanol evaporation. Investigators were blind to genotype or treatment of the mice being tested during all experiments. All animal studies were conducted with the approval of the Laboratory Animal Ethics Committee of Jinan University. Details of n values and statistics for different tests are listed in Supplementary Table 2 and 3.

*Nest building*

Nest building ability was tested according to a protocol described previously[^2^](#_ENREF_2). Approximately 1 h before the dark phase, the home cage of each mouse was changed with new bedding, and a pressed cotton square nesting material (Nestlet, Ancare) was provided. The nests built from Nestlets were evaluated the following morning (1 h after the dark phase), and rating scores 1–5 (where 5 is the best) were assigned according to the quality of the nests. All the untorn Nestlet pieces were combined and weighed.

*Open field test*

The open field test was performed in a white plastic box (40 × 40 × 40 cm), illuminated by overhead white lighting (~100 lux). Mice were placed in the center of the open field arena in the beginning of the test, and were allowed to freely explore the arena for 30 min. An overhead video camera was used to record the movement of the mice. Travel distance was analyzed using Topscan 3.0, Clever Sys Inc.

*Ultrasonic Vocalizations (USV) Recording*

Pups isolation calls are recorded as described previously [^3^](#_ENREF_3){, #210}. Briefly, for inducing isolation-induced USV, each pup was isolated from its dam and littermates at postnatal day 3, 6, 9 and 12. For each isolation, pups were placed into a glass beaker with new bedding in a foam box (temperature was maintained at 21± 1℃) for 5 min. Recording hardware (Avisoft UltraSound Gate 116 Hm with a high-quality condenser microphone) and software (Avisoft SASLab Pro Recorder) were from Avisoft Bioacoustics (16-bit format, 300 kHz sampling frequency to capture sound amplitude up to 150 kHz with a high quality). Number of calls, duration of total calls and average peak frequency for each pup were measured using the automatic parameter measurements function in the software (min duration: 2 ms; hold time: 10 ms) by filtering out the amplitude under 30 kHz.

*Pup retrieval*

Pup retrieval test was performed according to a published protocol with modification [^4^](#_ENREF_4). Briefly, virgin female mice, which had not given birth or were exposed to pups, were used. The subject mouse was introduced to a new home cage with a piece of Nestlet the night before test. On the next day, the mouse was briefly transferred to another experimental cage similar to the animal’s home cage, while three pups (1 to 3-day old) were randomly placed in three corners of the home cage that were not occupied by the nest. The subject mouse was then gently transferred back to the nest. Latency of pup retrieval was measured by the time spent to collect each pup to the nest. If the mouse failed to retrieve all pups within a 10 min session, the test was stopped and the time was recorded as 600 seconds.

*Three-chamber test*

Social approach and social novelty were tested in a rectangular three-chamber apparatus, and the size of each chamber is 20 cm (length) × 40 cm (width) × 20 cm (height). Chambers were separated by walls with bottom openings (5 × 5 cm). The test consisted of three different phases (habituation, social approach and social novelty). In the habituation phase, the mouse was placed in the middle chamber and was allowed to freely explore three chambers of the apparatus. After 10 min, the mouse was gently guided back to the middle chamber, and the doorways were blocked. In the social approach phase, a new C57BL/6 mouse (stranger #1) was placed under a wire mesh cup (8 cm in diameter and 10 cm in height) in one of the side chambers, while an identical empty cup was placed in the other side. After opening the doorways, the subject mouse was allowed to freely explore the stranger mouse or the empty cup (non-social object) for 10 min, and the doorways were blocked again. In the social novelty phase, a second C57BL/6 mouse (stranger #2) was introduced in the empty cup. After opening the doorways, the subject mouse was allowed to freely explore the new unfamiliar mouse or the familiar mouse for 10 min. The age and gender of the stranger mice were the same as the test mice, and they were habituated in the cup for 2–3 sections (15 min each) each day for 2 days before the test. The locations of stranger 1 and empty cup, or stranger 1 and stranger 2, were alternate between tests of different subjects. An overhead video camera was used to record the movement of the mice. Travel distance, number of entries into different chambers, the time spent in different chambers, and the time spent sniffing the stranger mice or the empty cup were analyzed using Topscan 3.0.

*Elevated zero-maze*

Anxiety-like behaviors were assessed using an elevated zero-maze as described previously [^5^](#_ENREF_5). The maze is elevated 60 cm from the floor, and has a 5.5 cm-wide circular corridor with a diameter of 53 cm. The corridor is divided into four quadrants of equal lengths: two opposing quadrants are the open sections which have 0.5 cm-high curbs to prevent falls; the other two opposing quadrants are closed sections which have walls of 14 cm in height. The illuminance of overhead white lighting is 40 lux. Each subject mouse was placed at one junction of open and closed sections, facing inside the closed one. Different junctions were chosen among subjects to avoid bias. The trial lasted 5 min for each mouse, and was recorded by an overhead video camera. Travel distance and velocity, duration and number of entries in the open and closed sections were analyzed by Topscan 3.0.

*Novel object recognition*

Recognition memory was assessed using novel object recognition test as described previously[^6^](#_ENREF_6). The test was performed in the same arena as open field (40 × 40 × 40 cm white plastic box) with an overhead white lighting (40 lux). First, the mice were placed into the arena to habituate for 10 min. After 1 h interval, the training session was conducted. Two identical objects (white plastic round bottle caps, 4 cm in diameter and 2.8 cm in height) were fixed in the box. The subject mouse was allowed to explore and recognize the objects for 10 min. 24 h after the training session, one of the identical objects was replaced by a novel object (a Lego structure, 4.6 × 2.2 × 3.5 cm), and the mouse was put back to explore the familiar and novel objects for 8 min. Total distance traveled and time spent in sniffing on each object was analyzed by Topscan 3.0.

*Y-maze working memory*

To determine spontaneous alternation behavior (an assay that measures spatial working memory), we tested the mice in a Y-maze with an overhead white lighting (40 lux) as described previously [^7^](#_ENREF_7)^,^ [^8^](#_ENREF_8). The apparatus is a capital Y-shape maze with three equal arms (34 cm long, 8 cm wide and 14 cm deep, and randomly designated as A, B, and C) separated by 120 degree angles. Each mouse was placed at the end of one of three arms and allowed to explore the maze for 8 min. An arm entry was considered when all four limbs left the center of the maze and were within the arm. Orders of entries into each of the A, B, and C arms were recorded. The number of alternations were counted according to the following definition: only a consecutive choice containing three different arms (i.e., ABC, ACB, CAB, etc.) was defined as an alternation. The percentage of spontaneous alternation was determined by the following formula: Alternation (%) = [number of actual alternations / maximum number of alternations in theory (total number of arm entries − 2)] x 100%. Normal distribution of the data were confirmed before statistical analysis.

*Y-maze spatial recognition memory*

Spatial recognition memory was tested in the same Y-maze as described above, but using a different protocol as reported previously[^9^](#_ENREF_9). The luminance of overhead white lighting is 40 lux and numerous visual cues were placed on the walls of the testing room. The test consisted of two trials. In the first trial, the mouse was put at the end of one arm (designated as the start arm). Only one of the other two arms was open (the familiar arm), and the third arm was blocked (the novel arm). The mouse was allowed to freely explore the start and familiar arm for 10 min. After a 2 h inter-trial interval, the novel arm was open, and the mouse was put back at the end of the start arm and was allowed to explore all three arms for 5 min. The choices of novel and familiar arm were alternate between tests of different subjects. Total distance, time spent and number of entries in different arms were analyzed by Topscan 3.0.

*Morris water maze*

Morris water maze test for assaying spatial learning was based on a standard method [^10^](#_ENREF_10). Briefly, a circular pool (120 cm in diameter, 45 cm in depth) was filled with water (maintained at 25℃) that contained titanium dioxide to reduce underwater visibility. Two principal axes perpendicular to each other were designated to divide the pool into four equal quadrants, and the intersection points of the axes with the pool edge were designated as North (N), South (S), East (E), and West (W) (shown in Supplementary Figure 5e). A round platform (10 cm in diameter, 30 cm in height) was placed at the center of the southwest quadrant (the SW or Target quadrant). Numerous visual objects were hung on walls to serve as extra-maze cues for spatial learning. The training phase lasted for 7 days, and the mice were given 3 trials (90 s each) per day with an inter-trial interval of 30 min. On Day 1, the escape platform was placed above the water and was visible to the mice. During each trial, the mice, facing the tank wall, were released from three start locations (N, NE [northeast] and E) in random order, and the time spent to locate the escape platform was recorded. If the mice did not find the platform within 90 s, they were gently guided to the platform, and the escape time was recorded as 90 s. Once on the platform, the mice were given 20 s to recognize and remember the cues. On Day 2–7, the tests were performed like the procedures in Day 1, except that the platform was submerged, 0.5 cm below the water surface. On Day 8 (24 h after the last trial), a probe test was given for 90 s with the platform removed. The mice, facing the tank wall, were placed from one of start positions. Time spent in different quadrants and the swim velocity were evaluated by Topscan 3.0.

*Marble burying*

The marble burying test was carried out as described previously [^11^](#_ENREF_11). In an experimental cage similar to the mice’s home cage, wood chip bedding was filled at a thickness of 5 cm. 20 black glass marbles (1.5 cm in diameter) were placed equidistantly in a 4 ×5 arrangement on the surface of bedding. The mouse was introduced into the test cage from one random corner and allowed for free exploration and digging for 20 min. The number of marbles buried (two thirds of the marble surface area was covered by bedding) was counted blindly to the genotype of the mouse.

*Self-Grooming*

Spontaneous grooming behavior was measured in an experimental cage similar to the mice’s home cage with thin bedding. Each mouse was given a period of 10 min for habitation, and then another 10 min for evaluate grooming behavior. A stopwatch was used to record accumulative time spent for grooming all body parts. Task scorer was blind to genotype of the mouse being tested.

**Electrophysiology**

Coronal slices from hippocampus (300 μm thick) were prepared from postnatal 3-month-old *Dock4* KO mice and their WT littermates using a tissue slicer (Vibratome 3000; Vibratome) in ice-cold dissection buffer containing the following (in mM): 212.7 sucrose, 3 KCl, 1.25 NaH_2_PO_4_, 3 MgCl_2_, 1 CaCl_2_, 26 NaHCO_3_, and 10 dextrose, bubbled with 95% O_2_/5% CO_2_. The slices were immediately transferred to ACSF at 35°C for 30 min before recordings. The recipe of ACSF was similar to the dissection buffer, except that sucrose was replaced with 124 mM NaCl, and the concentrations of MgCl_2_ and CaCl_2_ were changed to 1 mM and 2 mM, respectively. All recordings were performed at 31°C. Pyramidal cells in CA1 areas were identified visually under infrared differential interference contrast optics on the basis of their pyramidal somata and prominent apical dendrites.

To isolate AMPAR-mediated mEPSCs from pyramidal cells in CA1, 1 μM TTX, 20 μM bicuculline, and 100 μM D,L-APV were added to the ACSF (2 ml/min, 30 ± 1°C) which was continually bubbled with 95% O2/5% CO2. mEPSCs were recorded at a holding potential (Vh) of –70 mV using B700 amplifier (Axon Instruments), digitized at 2 kHz by a data acquisition board (National Instruments), and acquired using the Igor ProTM software (Wave Metrics). To isolate GABA receptor-mediated mIPSCs from pyramidal cells in CA1, 1 μM TTX, 20 μM CNQX, and 100 μM D,L-APV were added to the ACSF. mIPSCs were recorded at a holding potential (Vh) of –60 mV as inward current with the Cs-based internal solution consisting of the following (in mM): 120 CsCl, 8 NaCl, 2 EGTA, 10 HEPES, 5 QX‐314, 4 ATP, 10 Na‐phosphocreatine and 0.5 GTP, pH 7.4, at 270–290 mOsm. Acquired mEPSCs and mIPSCs were analyzed using the Mini Analysis ProgramTM (Synaptosoft). The threshold for detecting mEPSCs and mIPSCs was set at 3 times the Root Mean Square (RMS) noise. There was no significant difference in RMS noise between the WT and KO groups in both mEPSCs and mIPSCs. For the computation of kinetic parameters, 100–150 fully isolated events of mEPSCs and 400–500 fully isolated events of mIPSCs were averaged.

Evoked EPSCs were recorded by whole-cell voltage-clamp mode. A concentric bipolar stimulating electrode with a tip diameter of 125 μm (FHC) was placed in the stratum radiatum. The distance between stimulating and recording electrode was kept at 50–100 μm. Patch pipettes (2–4 mΩ) were filled with the internal solution consisting of the following (in mM): 120 Cs-methylsulfonate, 10 HEPES, 10 Na-phosphocreatine, 5 lidocaine N-ethyl bromide (QX-314), 4 ATP, 0.5 GTP, pH 7.2–7.3; the osmolarity of the solution was 270–285 mOsm. Only cells with series resistance <20 mΩ and input resistance >100 mΩ were studied. Cells were excluded if input resistance changed >15% or series resistance changed >10% over the experiment. Data were filtered at 3 kHz and digitized at 10 kHz using Igor Pro (WaveMetrics).

To measure E/I balance, evoked EPSC and evoked IPSC were recorded in the same cell in CA1 pyramidal cells. To isolate combined AMPA/NMDA receptor-mediated currents, neurons were held at the reversal potential for GABA_A_ receptor-mediated currents. To isolate GABA_A_ receptor-mediated currents, neurons were held at the reversal potential for AMPA/NMDA receptor-mediated currents. The reversal potentials for each neuron were determined by holding the membrane potential from −85 to −65 mV in 5 mV increments for GABA_A_ receptors and from -10 to +10 mV in 5 mV increments for AMPA/NMDA receptor. The average reversal potentials for EPSCs (WT: 2.9 ± 1.8 mV, n=22; KO; 1.9± 1.5 mV, n=22; P = 0.26) and IPSCs (WT: 74.1 ± 2.6 mV, n=22; KO: -77.6 ± 2.3 mV, n=22; P= 0.32) were not different between WT and KO mice, and at least 10 responses for the stimulus intensity to evoke the maximal amplitudes of EPSCs and IPSCs were averaged to measure the E/I ratio.

To examine the neurotransmitter release probability from presynaptic inputs in CA1 pyramidal neurons, paired-pulse ratio (PPR) of evoked EPSCs at -70 mV was measured at different inter-stimulus intervals (10, 20, 30, 50, 100, 200, 300, 500, 1000 and 2000 ms).

For input-output responses, AMPAR-EPSCs, recorded at –70 mV in the presence of APV(100 μM) and PTX (50 μm), and NMDAR-EPSCs, at +40 mV in the presence of CNQX (20 μM), PTX (50 μm) were evoked by a pulse electrical stimulus (0.1 ms width) with different stimulus intensities systematically (5, 10, 15, 20, 25, 30, 40, 60, and 80 μA). Inter-stimulus intervals were >15 s to minimize depression resulting from repetitive stimulation, and at least 10 responses for each intensity were averaged to measure the AMPAR-EPSCs and NMDAR-EPSCs. To obtain NMDAR-EPSC to AMPAR-EPSC ratio, AMPAR-EPSC was first recorded in ACSF solution (containing PTX) at –70 mV, and then the same cell was held at +40 mV to record NMDAR-EPSC in the presence of CNQX (10 μM) and PTX (50 μm) with the same stimulus pulse (0.1 ms, 50 μA); the ratio of the maximal amplitude of NMDAR-EPSPCs to AMPAR-EPSCs was defined as the NMDAR/AMPAR ratio. To evaluate the changes in functional fraction of NR2B at the synapses from WT or KO mice, a NR2B antagonist ifenprodil (3 μM) was applied and the proportion of the isolated NMDAR current was measured 20–30 min after application of the drug.

To induce LTP, a pairing protocol in whole-cell recording mode was applied [^12^](#_ENREF_12). In brief, conditioning stimulation consisted of 360 pulses at 2 Hz was paired with continuous postsynaptic depolarization (180 s) to 0 mV. To suppress excessive polysynaptic activity, picrotoxin (50 μm) was added in the recording bath, and the concentration of divalent cations was elevated to 4 mM Ca^2+^ and 4 mM Mg^2+^ to reduce recruitment of polysynaptic responses. A test pulse was delivered at 0.05 Hz to monitor baseline amplitude for 10 min before and for 25–35 min following paired stimulation. To calculate LTP, the EPSC amplitude was normalized to the mean baseline amplitude during 10 min baseline. Potentiation was defined as the mean normalized EPSC amplitude 25–40 min after paired stimulation. To examine whether LTP induced in KO and WT mice was NMDAR-dependent, APV at 100 μm were added into the ACSF. Data were acquired in an interleaved manner for LTP comparisons between KO and WT mice.

To induce LTD, a pairing protocol in whole-recording mode was also applied[^13^](#_ENREF_13). In brief, LFS at 1 Hz for 15 min (900 pulses) on presynaptic inputs paired with continuous postsynaptic depolarization at -40mV. For chemical NMDAR-LTD, 10 μm NMDA, an agonist of NMDAR was perfused into ACSF for 3 min to direct activate NMDAR.

Statistical significance was assessed with GraphPad Prism using unpaired t tests and one-way or two-way AVOVA. Comparison of distributions of mEPSC or mIPSC amplitude or frequence data was analyzed using two-sample Kolmogorov-Smirnov test. All the data reported in the text and figures represent the mean ± SEM.

**Golgi staining**

Mice were deeply anesthetized with sodium pentobarbital (60 mg/kg body weight) and perfused intracardially with PBS. Brains were removed and stained using a modified Golgi-Cox method (FD Rapid GolgiStainTM Kit) described previously [^14^](#_ENREF_14). Coronal brain sections of 200 μm thickness were obtained using a vibratome (Leica). These sections were collected on clean gelatin-coated microscope slides and submerged in ammonium hydroxide for 10 min, then washed with distilled water, dehydrated in successive baths of 50% (4 min), 75% (4 min), 90% (4 min) and 100% (3 × 5 min) ethanol, followed by 10 min in a xylene solution. Finally slides were mounted with neutral resin.

­­

**Biochemical measurement of synaptic proteins**

Hippocampi were cut out from 8 pairs of WT and KO mice brains and homogenized in an ice-cold solution buffer (0.5 M sucrose, 1 mM NaHCO_3_, 1 mM MgCl_2_, 0.5 mM CaCl_2_·2H_2_O, 10mM Na pyrophosphate) containing protease and phosphatase inhibitors (Selleck). After centrifugation at 1000 × g for 10 min to remove nuclei and insoluble debris, the supernatant was subjected to 10,000 × g centrifugation for 10 min to get a soft pellet. The pellet was then resuspended in a lysis buffer (1 mM NaHCO_3_, 0.32 M sucrose) containing protease and phosphatase inhibitors (Selleck), homogenized again, lysed for 30 min and centrifuged at 21,000 × g for 15 min. The pellet (crude synaptosome fraction containing synaptosomes and mitochondria) was dissolved in the solution buffer. Protein concentration was determined using a standard BSA method, and 10 µg of crude synaptic protein was separated by SDS-PAGE followed by Western blot analysis.

**Dock4 shRNA Lentivirus generation**

The Dock4 shRNA lentivirus used for infecting primary hippocampal neurons were prepared as described [^15^](#_ENREF_15). Briefly, pFUGW vectors containing Dock4 scramble (5'-GTGCATTGTACTGGTCTTT-3') or shRNA (5'-GAAGTTGTTCGGTTTCTCT-3') were transfected into HEK293T cells together with an HIV-1 packing vector 8.9 and a vesicular stomatitis virus glycoprotein (VSVg) envelope plasmid to generate lentiviral particles. The medium collected from the infected HEK293T cells was used to infect neurons.

**Primary hippocampal neuronal culture**

Primary hippocampal neurons were prepared and cultured from E18 Sprague-Dawley rat embryos as described previously [^15^](#_ENREF_15). Single cell suspension was seeded in 35-mm dishes coated with Poly-L-Lysine hydrobromide (PLL, 1 mg/ml) at a density of 5 × 10^5^ cells/35 mm dish. For measurement of glutamate receptor subunits, lentiviral shRNA or Rac1 were added to infect the neurons at 4 DIV, and corresponding protein expressions were detected at 9 DIV. MG132 (2 μM) was treated for 24 h before protein harvest when studying the involvement of proteasome-mediated protein degradation. For examination of global protein synthesis, lentiviral shRNA or Rac1 were added at 4 DIV, puromycin (1 mM) was added at 9 DIV for pulse labeling newly synthesized polypeptides for 30 min. Puromycin-labeled proteins were analyzed by western blotting. Alternatively, primary hippocampal neurons were cultured from 3 pairs of neonatal (P0) WT and KO mice. Puromycin was added at 9 DIV for studying global protein synthesis.

**Neuro-2a cell culture and transfection**

Neuro-2a cells (ATCC) were cultured in Minimum Essential Media (MEM; ThermoFishcer Scientific) supplemented with 10% Fetal Bovine Serum (FBS; ThermoFishcer Scientific). Plasmids were transfected into Neuro-2a cells using Lipofectamine LTX with Plus reagent (ThermoFishcer Scientific) as described previously [^15^](#_ENREF_15). After 24 h transfection, puromycin (1 mM) was added to cells for 30 min pulse labeling before protein harvest. For Rac1 inhibition, the cells were treated with NSC23766 for 2 h before harvest.

**Rac1 activity assay**

A Rac1 activity assay was performed using the Rac1/Cdc42 Activation Assay Kit (Merck Millipore) according to manufacturer’s instruction. Briefly, hippocampi were cut out from 6 pairs of WT and KO mice brains and homogenized with a Mg^2+^ Lysis/Wash Buffer containing 125 mM HEPES (pH 7.5), 750 mM NaCl, 5% Igepal CA-630, 50 mM MgCl_2_, 5 mM EDTA, 10% glycerol and protease inhibitors. Lysates were incubated with agarose beads conjugated with the p21 Rac/Cdc42 binding domain fused to GST (GST-PBD), which specifically binds to GTP-bound Rac1, at 4 °C for 60 min. The beads were washed three times with the Mg^2+^ Lysis/Wash Buffer. Bound Rac1-GTP proteins were then resuspended with sample buffer and subjected to Western blot analysis.

**Stereotaxic injection**

Mice were anesthetized with 1.25% tribromoethanol, and then fixed in a stereotaxic frame (RWD Life Science). Two craniotomies were performed at -2.30 mm (for male) or -2.15 mm (for female) anterior to bregma and ±1.50 mm (for male) or -1.35 mm (for female) lateral to the midline. For knockout Dock4 in the hippocampal CA1 region of *Dock4*^fl/fl^ mice, 200 nL adeno-associated virus (AAV) 2/9 serotype (pAOV-CAG-mCherry-T2A-Cre and pAAV-CAG-MCSmCherry-3FLAG, ObioTechnology) for each side was delivered through a glass capillary attached to a stereotaxic injector with a syringe pump (Harvard Apparatus) at 25 nL/min. For overexpression of Rac1 in the hippocampal CA1 region, 1000 nL lentivirus (pLenti-CMV-EGFP-P2A-3FLAG-Rac1 and pLenti-CMV-EGFP-P2A-MCS-3FLAG, ObioTechnology) was delivered at 100 nL/min. The injections were targeted to the center of hippocampus CA1 region at each side (AP -2.30 mm, LM ±1.50 mm, DV 1.23 mm for male mice and AP -2.15 mm, LM ±1.35 mm, DV 1.18 mm for female mice). The pipette was held in place for 10 min after injection and then slowly retracted. The incision was sutured. Animals were allowed to recover, and at least four weeks were given for optimal viral expression before behavior tests. Mice were subjected to the open field test, and the Three-chamber test on the following day. Electrophysiology was performed after behavior tests.

**Drug treatment**

D-cycloserine (DCS, Selleck) was dissolved in saline at a stock concentration of 2 mg/ml. A single dose of DCS (20 mg/kg body weight) was intraperitoneally (i.p.) administered to KO mice 90 min or 7 day before the Three-chamber test. Saline was administered at equal volume as a vehicle control. PF-4785774 (Sigma) was dissolved in DMSO at a stock concentration of 6 mg/ml, and was diluted into a solvent of 0.5:10:89.5 (v:v:v) DMSO:Tween-80:saline at a final concentration of 0.03 mg/ml. A single dose of PF-4785774 (0.3 mg/kg body weight) was i.p. administered to KO mice 90 min before the Three-chamber test. The solvent was administered at equal volume as a vehicle control.

**Reference**

1. Nishimura Y, Martin CL, Vazquez-Lopez A, Spence SJ, Alvarez-Retuerto AI, Sigman M *et al.* Genome-wide expression profiling of lymphoblastoid cell lines distinguishes different forms of autism and reveals shared pathways. *Human molecular genetics* 2007; **16**(14)**:** 1682-1698.

2. Deacon RM. Assessing nest building in mice. *Nature protocols* 2006; **1**(3)**:** 1117-1119.

3. Ferhat AT, Torquet N, Le Sourd AM, de Chaumont F, Olivo-Marin JC, Faure P *et al.* Recording Mouse Ultrasonic Vocalizations to Evaluate Social Communication. *Journal of visualized experiments : JoVE* 2016; (112).

4. Wang Z, Storm DR. Maternal Behavior is Impaired in Female Mice Lacking Type 3 Adenylyl Cyclase. *Neuropsychopharmacology* 2010; **36**(4)**:** 772-781.

5. Bell R, Duke AA, Gilmore PE, Page D, Begue L. Anxiolytic-like effects observed in rats exposed to the elevated zero-maze following treatment with 5-HT2/5-HT3/5-HT4 ligands. *Scientific reports* 2014; **4:** 3881.

6. Bevins RA, Besheer J. Object recognition in rats and mice: a one-trial non-matching-to-sample learning task to study 'recognition memory'. *Nature protocols* 2006; **1**(3)**:** 1306-1311.

7. Hughes RN. The value of spontaneous alternation behavior (SAB) as a test of retention in pharmacological investigations of memory. *Neuroscience and biobehavioral reviews* 2004; **28**(5)**:** 497-505.

8. Miedel CJ, Patton JM, Miedel AN, Miedel ES, Levenson JM. Assessment of Spontaneous Alternation, Novel Object Recognition and Limb Clasping in Transgenic Mouse Models of Amyloid-beta and Tau Neuropathology. *Journal of visualized experiments : JoVE* 2017; (123).

9. Dellu F, Mayo W, Cherkaoui J, Le Moal M, Simon H. A two-trial memory task with automated recording: study in young and aged rats. *Brain research* 1992; **588**(1)**:** 132-139.

10. Vorhees CV, Williams MT. Morris water maze: procedures for assessing spatial and related forms of learning and memory. *Nature protocols* 2006; **1**(2)**:** 848-858.

11. Angoa-Perez M, Kane MJ, Briggs DI, Francescutti DM, Kuhn DM. Marble burying and nestlet shredding as tests of repetitive, compulsive-like behaviors in mice. *Journal of visualized experiments : JoVE* 2013; (82)**:** 50978.

12. Li S, Wang L, Tie X, Sohya K, Lin X, Kirkwood A *et al.* Brief Novel Visual Experience Fundamentally Changes Synaptic Plasticity in the Mouse Visual Cortex. *The Journal of neuroscience : the official journal of the Society for Neuroscience* 2017; **37**(39)**:** 9353-9360.

13. Lee HK, Kameyama K, Huganir RL, Bear MF. NMDA induces long-term synaptic depression and dephosphorylation of the GluR1 subunit of AMPA receptors in hippocampus. *Neuron* 1998; **21**(5)**:** 1151-1162.

14. Gibb R, Kolb B. A method for vibratome sectioning of Golgi-Cox stained whole rat brain. *Journal of neuroscience methods* 1998; **79**(1)**:** 1-4.

15. Xiao Y, Peng Y, Wan J, Tang G, Chen Y, Tang J *et al.* The atypical guanine nucleotide exchange factor Dock4 regulates neurite differentiation through modulation of Rac1 GTPase and actin dynamics. *The Journal of biological chemistry* 2013; **288**(27)**:** 20034-20045.
