# Supplementary material for: Autism-like social deficit generated by Dock4 deficiency is rescued by restoration of Rac1 activity and NMDA receptor function
Source: Mol Psychiatry. 2019 Aug 6;26(5):1505–19. doi: 10.1038/s41380-019-0472-7 (PMC8159750; doi:10.1038/s41380-019-0472-7)
Supplement: Supplementary file 2 — Supplementary Table 1 [file 41380_2019_472_MOESM2_ESM.pdf]

Supplementary Table 1 *DOCK4* variations associated with autism spectrum disorder

DOCK4 variation[1]	SNP ID	Variant type	Allele change	Protein change	Phenotype	PUBMED ID	AUTHOR, YEAR
7:111796377 (A>C)	rs2217262	Intron_variant	c.37+49780T>G	/		19401682; 24599690	Maestrini E, et al. (2010); Liang S, et al. (2014)
7:111732571 (C>G)	rs7785891	Intron_variant	c.38-88385G>C	/		26176695	Warrier V, et al. (2015)
7:111584840 (A>G)		Intron_variant	c.844-26T>C	/		25363768	Iossifov I, et al. (2014)
7:111428810 (C>T)	rs200443099	Intron_variant[2]	c.3316-7G>A	/		23999528	Toma C, et al. (2013)
7:111381201 (T>C)		Synonymous_variant	A>G	/		28714951	Lim ET , et al. (2017)
7:111487098 (C>T)	rs2074130	Missense	c.2558G>A	p.R853H	Dyslexia	26184631	Shao S, et al. (2016)
7:111398732 (C>T)	rs373247688	Missense	c.4250G>A	p.R1417H		25363768	Iossifov I, et al. (2014)
7:111585776 (AAG>A)		Frame-shift	c.777CTT>T	Frame shift after 259 a.a., stop at 265 a.a.		25363768	Iossifov I, et al. (2014)
Deletion at 7:110663978-111257682		CNV	Exon 27-52 deleted	Frame shift after 945 a.a., stop at 947 a.a.	Dyslexia/poor reading ability	20346443	Pagnamenta AT, et al. (2010)
Deletion at 7:111777402 - 111865491 (on Assembly GRCh38)		CNV	Exon 23-35 deleted	Frame shift after 760 a.a., stop at 783 a.a	Microcephaly, characteristic face, intellectual disability, motor coordination deficits, language delay	30208311	Kushima et al. (2018)
Duplication at 7:110707538 - 111450107		CNV				19401682	Maestrini E, et al. (2010)
Deletion or duplication at 7q31.1 that spans *DOCK* region		CNV					Multiple studies[3]

[1]Positions are indicated according to Assembly GRCh37 unless otherwise stated

[2]This SNP causes start condon loss in one small transcript of *DOCK4* (ENST00000417165.1)

[3]7q31.1 CNVs (that spans *DOCK4* region) linked to ASD can be found in the following databases:
Autism database (AutDB): http://autism.mindspec.org/GeneDetail/DOCK4
AutismKB: http://db.cbi.pku.edu.cn/autismkb_v2/gene_evidence_detail.php?entrez_id=9732
SFARI (Simons Foundation Autism Research Initiative) Gene: https://gene.sfari.org/database/human-gene/DOCK4
