# Supplementary material for: Autism-like social deficit generated by Dock4 deficiency is rescued by restoration of Rac1 activity and NMDA receptor function
Source: Mol Psychiatry. 2019 Aug 6;26(5):1505–19. doi: 10.1038/s41380-019-0472-7 (PMC8159750; doi:10.1038/s41380-019-0472-7)
Supplement: Supplementary file 3 — Supplementary Table 2 [file 41380_2019_472_MOESM3_ESM.pdf]

Supplementary Table 2 Statistical details of behavior studies of WT, HET and KO mice

Test	# of Animal	Measurement	Note	Genotype	Average	s.e.m	*P* value (Statistical Test + *post hoc* Test)	Fig.
**Open field -male**	WT=11<br>HET=9<br>KO=11	Distance traveled (m)	-	WT	76.86	5.758	-	1f
			-	HET	73.77	5.107	-	
			-	KO	70.4	5.53	-	
**Open field -female**	WT=9<br>HET=11<br>HET=3<br>KO=8<br>KO=4		-	WT	77.75	9.942	-	
			-	HET	66.42	4.798	p<0.0001 (Unpaired *t* test)	
			Hyperactivity	HET	187.6	14.51		
			-	KO	73.33	5.952	p<0.0001 (Unpaired *t* test)	
			Hyperactivity	KO	265.3	40.6		
**USV**	WT=28<br>HET=39<br>KO=13	Number of calls / 5min	Day3	WT	113.7	17.86	-	1g
				HET	94.23	16.18	p=0.4820 (One-way ANOVA + B[1])	
				KO	199.2	45.4	p=0.0245 (One-way ANOVA + B)	
	WT=28<br>HET=41<br>KO=15		Day6	WT	332.9	39.83	-	
				HET	355.3	38.74	p=0.7107 (One-way ANOVA + B)	
				KO	422.1	75.84	p=0.2587 (One-way ANOVA + B)	
	WT=29<br>HET=38<br>KO=15		Day9	WT	271.6	40.63	-	
				HET	258.7	28.34	p=0.8034 (One-way ANOVA + B)	
				KO	362.4	68.21	p=0.1753 (One-way ANOVA + B)	
	WT=26<br>HET=38<br>KO=13		Day12	WT	121.9	26.18	-	
				HET	106.8	20.34	p=0.7034 (One-way ANOVA + B)	
				KO	241.5	69.41	p=0.0260 (One-way ANOVA + B)	
	WT=28	Total call duration (s)	Day3	WT	2.181	0.3932	-	S6a

	HET=39<br>KO=13			HET	2.072	0.4378	p=0.8922 (One-way ANOVA + B)	
				KO	5.84	1.594	p=0.0011 (One-way ANOVA + B)	
	WT=28<br>HET=41<br>KO=15		Day6	WT	8.009	1.283	-	
				HET	7.774	1.184	p=0.9047 (One-way ANOVA + B)	
				KO	11.65	2.754	p=0.1567 (One-way ANOVA + B)	
	WT=29<br>HET=38<br>KO=15		Day9	WT	6.945	1.507	-	
				HET	5.959	0.8831	p=0.5851 (One-way ANOVA + B)	
				KO	10.4	2.45	p=0.1402 (One-way ANOVA + B)	
	WT=26<br>HET=38<br>KO=13		Day12	WT	2.159	0.5424	-	
				HET	1.679	0.3727	p=0.5921 (One-way ANOVA + B)	
				KO	4.987	1.831	p=0.0202 (One-way ANOVA + B)	
	WT=28<br>HET=39<br>KO=13	Peak frequency (kHz)	Day3	WT	87.4	4.928	-	S6b
				HET	87.28	2.768	p=0.9795 (One-way ANOVA + B)	
				KO	98.26	2.138	p=0.1079 (One-way ANOVA + B)	
	WT=28<br>HET=41<br>KO=15		Day6	WT	88.48	1.492	-	
				HET	87.88	0.9857	p=0.7622 (One-way ANOVA + B)	
				KO	89.52	3.12	p=0.6884 (One-way ANOVA + B)	
	WT=29<br>HET=38<br>KO=15		Day9	WT	90.2	2.346	-	
				HET	93.89	1.355	p=0.1593 (One-way ANOVA + B)	
				KO	93.68	2.844	p=0.3013 (One-way ANOVA + B)	
	WT=26<br>HET=38<br>KO=13		Day12	WT	86.34	5.065	-	
				HET	82.78	4.71	p=0.5851 (One-way ANOVA + B)	
				KO	93.24	1.677	p=0.4291 (One-way ANOVA + B)	
**Nesting - male**	WT=27	Score (0-5)	-	WT	3.87	0.2226	-	S6d

	HET=12<br>KO=19		-	HET	4	0.2752	p>0.9999 (Kruskal-Wallis test +D[2])	
			-	KO	3.237	0.3138	p=0.1314 (Kruskal-Wallis test +D)	
		untorn nestlets (g)	-	WT	0.5911	0.1587	-	S6e
			-	HET	0.3442	0.2121	p=0.9050 (One-way ANOVA + B)	
			-	KO	0.8663	0.2706	p=0.6657 (One-way ANOVA + B)	
**Nesting - female**	WT=23<br>HET=10<br>KO=20	Score (0–5)	-	WT	3.739	0.2152	-	S6d
			-	HET	3.85	0.2587	p>0.9999 (Kruskal-Wallis test +D)	
			-	KO	3.75	0.2099	p>0.9999 (Kruskal-Wallis test +D)	
		untorn nestlets (g)	-	WT	0.5383	0.1678	-	S6e
			-	HET	0.189	0.1516	p=0.3919 (One-way ANOVA + B)	
			-	KO	0.408	0.1489	p>0.9999 (One-way ANOVA + B)	
**Pup retrieval**	WT=13<br>HET=12<br>KO=9	Latency (s)	First pup	WT	185.9	48.5	-	1h
				HET	246.8	58.26	p=0.4351 (One-way ANOVA + B)	
				KO	434	67.98	p=0.0057 (One-way ANOVA + B)	
			Last pup	WT	340.1	52.74	-	
				HET	337.8	62.55	p=0.9754 (One-way ANOVA + B)	
				KO	516.3	43.48	p=0.0377 (One-way ANOVA + B)	
**Three chamber - male**	WT=15<br>HET=13<br>KO=14	Sniffing time (s) - Social approach	Mouse (S1)	WT	120.9	11.78	p=0.0024 (Unpaired *t* test); p=0.0004(Two-way ANOVA + B[3])	1j
			Empty (E)	WT	75.57	6.777		
			Mouse (S1)	HET	122.3	9.505	p<0.0001 (Unpaired *t* test); p<0.0001 (Two-way ANOVA + B)	
			Empty (E)	HET	58.41	5.481		
			Mouse (S1)	KO	104.2	8.898	p<0.0001 (Unpaired *t* test); p=0.0001 (Two-way ANOVA + B)	
			Empty (E)	KO	52.49	4.504		
		Sniffing time (s) -	Familial (S1)	WT	51.14	6.551	p=0.0031 (Unpaired *t* test); p=0.0032	1l

		Social novelty	Unfamiliar (S2)	WT	97.52	12.71	(Two-way ANOVA + B)	
			Familial (S1)	HET	60.59	8.576	p=0.0116 (Unpaired *t* test); p=0.0518 (Two-way ANOVA + B)	
			Unfamiliar (S2)	HET	96.28	9.838		
			Familial (S1)	KO	54.65	8.703	p=0.5017 (Unpaired *t* test); p>0.9999 (Two-way ANOVA + B)	
			Unfamiliar (S2)	KO	64.7	11.91		
		Duration in chamber (s) - Social approach	Mouse (S1)	WT	292.7	12.44	p<0.0001 (One-way ANOVA + B); p=0.0005 (Two-way ANOVA + D[4])	S6f
			Empty (E)	WT	219.3	12.1		
			Center (C)	WT	87.03	10.28	-	
			Mouse (S1)	HET	320.7	12.59	p<0.0001 (One-way ANOVA + B); p=0.0001 (Two-way ANOVA + D)	
			Empty (E)	HET	189.7	11.65		
			Center (C)	HET	88.36	8.241	-	
			Mouse (S1)	KO	317.8	17.1	p<0.0001 (One-way ANOVA + B); p=0.0001 (Two-way ANOVA + D)	
			Empty (E)	KO	200.9	19.62		
			Center (C)	KO	80.1	6.973	-	
		Duration in chamber (s) - Social novelty	Mouse (S1)	WT	214.4	15.22	p=0.0015 (One-way ANOVA + B); p=0.0355 (Two-way ANOVA + D)	S6h
			Mouse (S2)	WT	284.2	17.88		
			Center (C)	WT	94.88	8.352	-	
			Mouse (S2)	HET	237	18.81	p=0.2157 (One-way ANOVA + B); p=0.8552 (Two-way ANOVA + D)	
			Empty (E)	HET	265	18.05		
			Center (C)	HET	94.45	7.892	-	
			Mouse (S2)	KO	267.3	26.57	p=0.4806 (One-way ANOVA + B); p=0.9281 (Two-way ANOVA + D)	
			Empty (E)	KO	244.2	27.67		
			Center (C)	KO	87.45	10.56	-	
**Three chamber -**	WT=13	Sniffing time (s) -	Mouse (S1)	WT	100.4	10.28	p=0.012 (Unpaired *t* test); p=0.0096	1k

**female**	HET=13<br>KO=12	Social approach	Empty (E)	WT	68.36	5.962	(Two-way ANOVA + B)	
			Mouse (S1)	HET	80.45	5.678	p=0.0082 (Unpaired *t* test); p=0.0416 (Two-way ANOVA + B)	
			Empty (E)	HET	53.95	7.235		
			Mouse (S1)	KO	84.31	8.518	p=0.0287 (Unpaired *t* test); p=0.0774 (Two-way ANOVA + B)	
			Empty (E)	KO	59.43	6.351		
		Sniffing time (s) - Social novelty	Familial (S1)	WT	48.84	4.727	p=0.0017 (Unpaired *t* test); p=0.0021 (Two-way ANOVA + B)	1m
			Unfamiliar (S2)	WT	82.05	8.105		
			Familial (S1)	HET	45.55	7.602	p=0.4077 (Unpaired *t* test); p>0.9999 (Two-way ANOVA + B)	
			Unfamiliar (S2)	HET	53.12	4.788		
			Familial (S1)	KO	43.79	7.543	p=0.4379 (Unpaired *t* test); p>0.9999 (Two-way ANOVA + B)	
			Unfamiliar (S2)	KO	51.78	6.721		
		Duration in chamber (s) - Social approach	Mouse (S1)	WT	270.1	15.04	p=0.012 (One-way ANOVA + B); p=0.031 (Two-way ANOVA + D)	S6g
			Empty (E)	WT	216.9	15.55		
			Center (C)	WT	112.6	11.94	-	
			Mouse (S1)	HET	261.2	9.544	p=0.0044 (One-way ANOVA + B); p=0.0075 (Two-way ANOVA + D)	
			Empty (E)	HET	196	17.37		
			Center (C)	HET	142.7	17.28	-	
			Mouse (S1)	KO	271.2	19.14	p=0.0683 (One-way ANOVA + B); p=0.0698 (Two-way ANOVA + D)	
			Empty (E)	KO	222.9	23.2		
			Center (C)	KO	105.7	8.905	-	
		Duration in chamber (s) - Social novelty	Mouse (S1)	WT	186	14.97	p<0.0001 (One-way ANOVA + B); p=0.0001 (Two-way ANOVA + D)	S6i
			Empty (E)	WT	291.2	19.24		
			Center (C)	WT	122.2	13.18	-	
			Mouse (S1)	HET	209	19.53	p=0.0621 (One-way ANOVA + B);	

			Empty (E)	HET	256.9	18.97	p=0.0779 (Two-way ANOVA + D)	
			Center (C)	HET	133.5	13.77	-	
			Mouse (S1)	KO	234.7	17.09	p=0.6708 (One-way ANOVA + B); p=0.8841 (Two-way ANOVA + D)	
			Empty (E)	KO	244.7	20.15		
			Center (C)	KO	119.8	11.07	-	
**Elevated zero maze - male**	WT=19 HET=17 KO=15	Distance traveled (m)	-	WT	8.873	0.8724	-	2a
			-	HET	7.865	0.4897	p=0.2783 (One-way ANOVA + B)	
			-	KO	6.209	0.4265	p=0.0073 (One-way ANOVA + B)	
		Distance traveled (m)- closed sections	-	WT	7.487	0.5972	-	S7a
			-	HET	6.994	0.3894	p=0.4582 (One-way ANOVA + B)	
			-	KO	5.624	0.3366	p=0.0088 (One-way ANOVA + B)	
		Distance traveled (m)- open sections	-	WT	1.386	0.3105	-	S7c
			-	HET	0.8708	0.1562	p=0.1125 (One-way ANOVA + B)	
			-	KO	0.585	0.1395	p=0.0189 (One-way ANOVA + B)	
		Duration in sections (%)	Open	WT	12.29	1.477	-	2b
				HET	10.76	1.443	p=0.4724 (One-way ANOVA + B)	
				KO	7.579	1.717	p=0.0365 (One-way ANOVA + B)	
		Number of entries	Open	WT	10.68	1.508	-	2c
				HET	7.647	1.029	p=0.0831 (One-way ANOVA + B)	
				KO	5.8	0.9817	p=0.0084 (One-way ANOVA + B)	
		Velocity (mm/s) - closed sections	-	WT	29.17	2.822	-	S7b
			-	HET	26.46	1.686	p=0.1068 (One-way ANOVA + B)	
			-	KO	20.56	1.493	p=0.0251 (One-way ANOVA + B)	
		Velocity (mm/s) -	-	WT	31.84	3.568	-	S7d

		open sections	-	HET	25.14	2.226	p=0.1068 (One-way ANOVA + B)	
			-	KO	22.08	2.636	p=0.0086 (One-way ANOVA + B)	
**Elevated zero maze - female**	WT=22 HET=13 KO=11	Distance traveled (m)	-	WT	8.905	0.6067	-	2a
			-	HET	9.154	0.7395	p=0.7915 (One-way ANOVA + B)	
			-	KO	6.578	0.6806	p=0.0228 (One-way ANOVA + B)	
		Distance traveled (m)- closed section	-	WT	7.387	0.3826	-	S7a
			-	HET	7.863	0.5552	p=0.5621 (One-way ANOVA + B)	
			-	KO	5.575	0.5168	p=0.0106 (One-way ANOVA + B)	
		Distance traveled (m)- open section	-	WT	1.518	0.2882	-	S7c
			-	HET	1.291	0.239	p=0.4632 (One-way ANOVA + B)	
			-	KO	1.003	0.2317	p=0.2164 (One-way ANOVA + B)	
		Duration in sections (%)	Open	WT	14.19	2.088	-	2b
				HET	11.82	1.601	p=0.4543 (One-way ANOVA + B)	
				KO	11.79	2.639	p=0.4361 (One-way ANOVA + B)	
		Number of entries	Open	WT	10.68	1.573	-	2c
				HET	9.308	1.337	p=0.5243 (One-way ANOVA + B)	
				KO	6.636	1.309	p=0.0804 (One-way ANOVA + B)	
		Velocity (mm/s) - closed sections	-	WT	29.69	2.213	-	S7b
			-	HET	30.12	2.427	p=0.3657 (One-way ANOVA + B)	
			-	KO	21.5	2.237	p=0.0219 (One-way ANOVA + B)	
		Velocity (mm/s) - open sections	-	WT	31.23	2.371	-	S7d
			-	HET	34.9	3.073	p=0.3657 (One-way ANOVA + B)	
			-	KO	30.25	3.8	p=0.8177 (One-way ANOVA + B)	
**Novel object**	WT=13	Preference for object	Familial	WT	34.41	3.644	p<0.0001 (Unpaired *t* test)	2d

**recognition - male**	HET=9<br>KO=12	(%) – 24h	Novel	WT	65.59	3.644		
			Familial	HET	41.94	2.58	p=0.0004 (Unpaired *t* test)	
			Novel	HET	58.06	2.58		
			Familial	KO	39.08	4.344	p=0.0018 (Unpaired *t* test)	
			Novel	KO	60.92	4.344		
**Novel object recognition - female**	WT=17<br>HET=10<br>KO=8	Preference for object (%) – 24h	Familial	WT	36.25	3.171	p<0.0001 (Unpaired *t* test)	2d
			Novel	WT	63.75	3.171		
			Familial	HET	32.96	4.385	p<0.0001 (Unpaired *t* test)	
			Novel	HET	67.04	4.385		
			Familial	KO	45.67	3.706	p=0.1204 (Unpaired *t* test)	
			Novel	KO	54.33	3.706		
**Y-maze - male (working memory)**	WT=19<br>HET=17<br>KO=15	Alternation (%)	-	WT	59.84	2.068	-	2e
			-	HET	58	1.961	p=0.4918 (One-way ANOVA + B)	
			-	KO	53.97	1.566	p=0.0374 (One-way ANOVA + B)	
		Number of arms entries	-	WT	39.63	3.454	-	2f
			-	HET	41.88	3.375	p=0.6603 (One-way ANOVA + B)	
			-	KO	53.8	4.351	p=0.0098 (One-way ANOVA + B)	
**Y-maze - female (working memory)**	WT=23<br>HET=13<br>KO=12	Alternation (%)	-	WT	56.4	1.408	-	2e
			-	HET	59.54	2.322	p=0.2740 (One-way ANOVA + B)	
			-	KO	56.8	2.956	p=0.8923 (One-way ANOVA + B)	
		Number of arms entries	-	WT	42.74	2.709	-	2f
			-	HET	43.15	3.901	p=0.9324 (One-way ANOVA + B)	
			-	KO	42.75	4.553	p=0.9983 (One-way ANOVA + B)	
**Y-maze - male**	WT=10	Time in arm (%)	Novel	WT	60.01	4.764	p=0.0082 (Unpaired *t* test)	2g

**(spatial memory)**	HET=11<br>KO=7		Familiar	WT	39.99	4.764		
			Novel	HET	69.29	2.668	p<0.0001 (Unpaired *t* test)	
			Familiar	HET	30.71	2.668		
			Novel	KO	47.91	7.605	p=0.7046 (Unpaired *t* test)	
			Familiar	KO	52.09	7.605		
		Number of arm entries (%)	Novel	WT	40.31	2.147	p<0.0001 (Unpaired *t* test)	2h
			Familiar	WT	24.3	2.113		
			Novel	HET	39.74	2.332	p<0.0001 (Unpaired *t* test)	
			Familiar	HET	21.75	1.867		
			Novel	KO	34.88	2.515	p=0.1301 (Unpaired *t* test)	
			Familiar	KO	29.9	1.752		
**Y-maze - female (spatial memory)**	WT=10<br>HET=8<br>KO=10	Time in arm (%)	Novel	WT	59.15	5.327	p=0.0258 (Unpaired *t* test)	2g
			Familiar	WT	40.85	5.327		
			Novel	HET	53.61	3.645	p=0.1828 (Unpaired *t* test)	
			Familiar	HET	46.39	3.645		
			Novel	KO	64.87	2.876	p<0.0001 (Unpaired *t* test)	
			Familiar	KO	35.13	2.876		
		Number of arm entries (%)	Novel	WT	37.63	3.284	p=0.0416 (Unpaired *t* test)	2h
			Familiar	WT	28.26	2.727		
			Novel	HET	39.83	1.07	p<0.0001 (Unpaired *t* test)	
			Familiar	HET	25.93	1.805		
			Novel	KO	36.99	1.415	p<0.0001 (Unpaired *t* test)	
			Familiar	KO	25.15	1.503		
**Morris water**	WT=17	Latency to platform	Day1	WT	67.43	4.566	-	S7f

**maze**	HET=16<br>KO=17	(s)		HET	64.71	5.23	p=0.6955 (One-way ANOVA + B);<br>p=0.9996 (Two-way ANOVA + D)	
				KO	70.39	4.807	p=0.6657 (One-way ANOVA + B);<br>p=0.9995 (Two-way ANOVA + D)	
			Day2	WT	64.41	5.464	-	
				HET	53.29	7.073	p=0.1936 (One-way ANOVA + B);<br>p=0.8661 (Two-way ANOVA + D)	
				KO	65.04	5.222	p=0.9401 (One-way ANOVA + B);<br>p=0.9999 (Two-way ANOVA + D)	
			Day3	WT	52.9	5.852	-	
				HET	47.33	5.75	p=0.4889 (One-way ANOVA + B);<br>p=0.9990 (Two-way ANOVA + D)	
				KO	58.84	5.23	p=0.4536 (One-way ANOVA + B);<br>p=0.9988 (Two-way ANOVA + D)	
			Day4	WT	46.75	7.838	-	
				HET	43.31	7.387	p=0.7304 (One-way ANOVA + B);<br>p=0.9997 (Two-way ANOVA + D)	
				KO	52.35	5.484	p=0.5680 (One-way ANOVA + B);<br>p=0.9940 (Two-way ANOVA + D)	
			Day5	WT	43.27	7.49	-	
				HET	38.96	6.011	p=0.6389 (One-way ANOVA + B);<br>p=0.9996 (Two-way ANOVA + D)	
				KO	46.25	5.562	p=0.7420 (One-way ANOVA + B);<br>p=0.9991 (Two-way ANOVA + D)	
			Day6	WT	44.25	5.462	-	
				HET	33.25	5.233	p=0.1670 (One-way ANOVA + B);<br>p=0.9810 (Two-way ANOVA + D)	
				KO	45.04	5.794	p=0.9195 (One-way ANOVA + B);<br>p=0.9995 (Two-way ANOVA + D)	
			Day7	WT	37.1	6.079	-	

				HET	31.85	5.335	p=0.5063 (One-way ANOVA + B);<br>p=0.9996 (Two-way ANOVA + D)	
				KO	44.16	5.023	p=0.3645 (One-way ANOVA + B);<br>p=0.9416 (Two-way ANOVA + D)	
		Duration in quadrant (%)	Target	WT	43.73	4.025	p<0.0001 (Unpaired *t* test)	S7g
			Opposite	WT	13.48	2.488		
			Target	HET	33.78	2.611	p=0.0003 (Unpaired *t* test)	
			Opposite	HET	18.62	2.57		
			Target	KO	34.73	2.995	p<0.0001 (Unpaired *t* test)	
			Opposite	KO	16.44	2.222		
		Swimming speed (s)	-	WT	172.1	11.17	-	S7h
			-	HET	182.5	8.799	p=0.4935 (One-way ANOVA + B)	
			-	KO	179.7	11.41	p=0.6133 (One-way ANOVA + B)	
**Marble burying**	WT=25<br>HET=25<br>KO=27	Number of marble buried	-	WT	11.11	1.158	-	S5b
			-	HET	10.92	1.283	p>0.9999 (One-way ANOVA + B)	
			-	KO	11.93	1.081	p>0.9999 (One-way ANOVA + B)	
**Grooming**	WT=7<br>HET=6<br>KO=8	Duration of grooming (s)	-	WT	30.71	6.494	-	S5c
			-	HET	26.33	7.306	p>0.9999 (One-way ANOVA + B)	
			-	KO	28.25	4.527	p>0.9999 (One-way ANOVA + B)	

[1]One-way ANOVA + B: One-way ANOVA with Bonferroni's Multiple Comparison Test
[2]Kruskal-Wallis test +D: Kruskal-Wallis test with Dunn's Multiple Comparison Test
[3]Two-way ANOVA + B: One-way ANOVA with Bonferroni's Multiple Comparison Test
[4]Two-way ANOVA + D: One-way ANOVA with Dunn's Multiple Comparison Test
