# Supplementary material for: Autism-like social deficit generated by Dock4 deficiency is rescued by restoration of Rac1 activity and NMDA receptor function
Source: Mol Psychiatry. 2019 Aug 6;26(5):1505–19. doi: 10.1038/s41380-019-0472-7 (PMC8159750; doi:10.1038/s41380-019-0472-7)
Supplement: Supplementary file 4 — Supplementary Table 3 [file 41380_2019_472_MOESM4_ESM.pdf]

Supplementary Table 3 Statistical details of behavior studies of virus injection or drug treatment in KO mice

**Test**	**# of Animal**	**Measurement**	**Treatment**	**Note**	**Average**	**s.e.m**	***P* value**	**Fig.**
**Three chamber (adeno-associated virus injection)**	$Dock4^{fl/fl}$+Vector =11 $Dock4^{fl/fl}$+Cre=8	Distance traveled (m) - Habitation	AAV-mCherry inj.	-	31.62	1.631	p=0.9544 (Unpaired *t* test)	S8c
			AAV-Cre inj.	-	31.75	1.339		
		Duration in chamber - Social approach	AAV-mCherry inj.	Mouse (S1)	286.1	11.03	p<0.0001 (One-way ANOVA + B[1]); p=0.0014 (Two-way ANOVA + D[2])	S8d
				Empty (E)	212.1	10.21		
				Center (C)	101.8	6.8	-	
			AAV-Cre inj.	Mouse (S1)	323.6	26.28	p<0.0001 (One-way ANOVA + B); p<0.0001 (Two-way ANOVA + D)	
				Empty (E)	167.4	21.78		
				Center (C)	109.1	12.86	-	
		Duration in chamber - Social novelty	AAV-mCherry inj.	Familial (S1)	209.1	14.08	p<0.0001 (One-way ANOVA + B); p=0.0087 (Two-way ANOVA + D)	S8e
				Unfamiliar (S2)	287.1	11.92		
				Center (C)	103.8	3.632	-	
			AAV-Cre inj.	Familial (S1)	239.9	33.47	p=0.5896 (One-way ANOVA + B); p>0.9999 (Two-way ANOVA + D)	
				Unfamiliar (S2)	261	32.37		
				Center (C)	99.2	7.729	-	
		Sniffing time (s) - Social approach	AAV-mCherry inj.	Mouse (S1)	94.74	5.859	p=0.0258 (Unpaired *t* test); p=0.0467(Two-way ANOVA + B[3])	3c
				Empty (E)	70.09	8.392		
			AAV-Cre inj.	Mouse (S1)	113.9	9.465	p=0.0006 (Unpaired *t* test); p=0.0002(Two-way ANOVA + B)	
				Empty (E)	59.3	8.032		
		Sniffing time (s) - Social novelty	AAV-mCherry inj.	Familial (S1)	52.33	6.37	p=0.001 (Unpaired *t* test); p=0.0061(Two-way ANOVA + B)	3d
				Unfamiliar (S2)	86.27	6.127		
			AAV-Cre inj.	Familial (S1)	64.28	13.22	p=0.2508 (Unpaired *t* test);	

				Unfamiliar (S2)	84.14	7.031	p=0.2409(Two-way ANOVA + B)	
**Open field**	KO+Vector=8<br>KO+Rac1=7	Distance traveled (m)	LV-GFP inj.	-	88.84	6.181	p=0.4448 (Unpaired *t* test)	S13a
			LV-Rac1 inj.	-	95.47	5.543		
**Three chamber (lentivirus injection)**	KO+Vector=8<br>KO+Rac1=7	Duration in chamber - Social approach	LV-GFP inj.	Mouse (S1)	281.2	10.36	p=0.0003 (One-way ANOVA + B);<br>p=0.0045 (Two-way ANOVA + B)	S13b
				Empty (E)	226.7	10.06		
				Center (C)	92.1	5.896	-	
			LV-Rac1 inj.	Mouse (S1)	308.2	13.27	p<0.0001 (One-way ANOVA + B);<br>p<0.0001 (Two-way ANOVA + B)	
				Empty (E)	206.3	16.6		
				Center (C)	85.3	10.63	-	
		Duration in chamber - Social novelty	LV-GFP inj.	Familial (S1)	236.7	12.68	p=0.1673 (One-way ANOVA + B);<br>p=0.7900 (Two-way ANOVA + B)	S13c
				Unfamiliar (S2)	264.8	17.43		
				Center (C)	98.37	10.81	-	
			LV-Rac1 inj.	Familial (S1)	210.6	20.47	p=0.0055 (One-way ANOVA + B);<br>p=0.0057 (Two-way ANOVA + B)	
				Unfamiliar (S2)	289.5	22.24		
				Center (C)	99.92	5.068	-	
		Sniffing time (s) - Social approach	LV-GFP inj.	Mouse (S1)	107.3	4.932	p=0.0003 (Unpaired *t* test);<br>p=0.0015(Two-way ANOVA + B)	6i
				Empty (E)	68.46	6.365		
			LV-Rac1 inj.	Mouse (S1)	103.9	8.609	p=0.0060 (Unpaired *t* test);<br>p=0.0010(Two-way ANOVA + B)	
				Empty (E)	60.41	9.863		
		Sniffing time (s) - Social novelty	LV-GFP inj.	Familial (S1)	61.15	10.99	p=0.5157 (Unpaired *t* test);<br>p=0.8951(Two-way ANOVA + B)	6j
				Unfamiliar (S2)	71.05	9.982		
			LV-Rac1 inj.	Familial (S1)	44.88	2.959	p=0.0061 (Unpaired *t* test);<br>p=0.0314(Two-way ANOVA + B)	
				Unfamiliar (S2)	80.35	10.27		
**Three**	KO+Vector=10	Duration in	Saline - 90min	Familial (S1)	229.5	25.66	p=0.5799 (One-way ANOVA + B);	S13e

**chamber (drug treatment)- DCS**	KO+DCS=10	chamber - Social novelty		Unfamiliar (S2)	246.2	25.15	p>0.9999 (Two-way ANOVA + B)	
				Center (C)	124.2	6.324	-	
			DCS - 90min 20mg/kg	Familial (S1)	179.2	18.03	p=0.0002 (One-way ANOVA + B); p=0.0003 (Two-way ANOVA + B)	
				Unfamiliar (S2)	305.4	23.74		
				Center (C)	115.2	18.68	-	
		Sniffing time (s) - Social novelty	Saline - 90min	Familial (S1)	45.84	10.07	p=0.3288 (Unpaired *t* test); p=0.4964(Two-way ANOVA + B)	6m
				Unfamiliar (S2)	59.06	8.494		
			DCS - 90min 20mg/kg	Familial (S1)	37.51	4.947	p=0.0093 (Unpaired *t* test); p=0.0528(Two-way ANOVA + B)	
				Unfamiliar (S2)	63.6	7.469		
		Duration in chamber - Social novelty	Saline - 7days	Familial (S1)	236.6	24.47	p=0.7473 (One-way ANOVA + B); p>0.9999 (Two-way ANOVA + B)	S13f
				Unfamiliar (S2)	245.1	18.66		
				Center (C)	117.8	7.501	-	
			DCS -7days 20mg/kg	Familial (S1)	242.1	20.3	p=0.8335 (One-way ANOVA + B); p>0.9999 (Two-way ANOVA + B)	
				Unfamiliar (S2)	235.5	28.07		
				Center (C)	122.4	16.92	-	
		Sniffing time (s) - Social novelty	Saline - 7days	Familial (S1)	42.79	5.992	p=0.5034 (One-way ANOVA + B); p>0.9999 (Two-way ANOVA + B)	S13g
				Unfamiliar (S2)	49.74	8.231		
			DCS -7days 20mg/kg	Familial (S1)	43.1	5.694	p=0.7124 (One-way ANOVA + B); p>0.9999 (Two-way ANOVA + B)	
				Unfamiliar (S2)	46.49	7.012		
**Three chamber (drug treatment)- PF-4778574**	KO+Vector=8 KO+PF-4778574 =9	Duration in chamber - Social novelty	Saline - 90min	Familial (S1)	242.6	25.68	p=0.9117 (Unpaired *t* test); p>0.999 (Two-way ANOVA + B)	S13i
				Unfamiliar (S2)	239.5	18.59		
				Center (C)	117.9	10.85	-	
			PF-4778574 - 90min 0.3mg/kg	Familial (S1)	253.2	16.98	p=0.3685 (Unpaired *t* test); p=0.8420 (Two-way ANOVA + B)	
				Unfamiliar (S2)	234.4	15.49		

				Center (C)	111.9	10.04	-	
		Sniffing time (s) - Social novelty	Saline - 90min	Familial (S1)	41.97	7.564	p=0.5215 (Unpaired *t* test); p>0.9999 (Two-way ANOVA + B)	S13j
				Unfamiliar (S2)	48.96	7.467		
			PF-4778574 - 90min 0.3mg/kg	Familial (S1)	38.3	9.239	p=0.4387 (Unpaired *t* test); p=0.7592(Two-way ANOVA + B)	
				Unfamiliar (S2)	48.97	9.75		

[1]One-way ANOVA + B: One-way ANOVA with Bonferroni’s Multiple Comparison Test
[2]Two-way ANOVA + D: One-way ANOVA with Dunn’s Multiple Comparison Test
[3]Two-way ANOVA + B: One-way ANOVA with Bonferroni’s Multiple Comparison Test
