# Supplementary material for: Autism-like social deficit generated by Dock4 deficiency is rescued by restoration of Rac1 activity and NMDA receptor function
Source: Mol Psychiatry. 2019 Aug 6;26(5):1505–19. doi: 10.1038/s41380-019-0472-7 (PMC8159750; doi:10.1038/s41380-019-0472-7)
Supplement: Supplementary file 5 — Supplementary Table 4 [file 41380_2019_472_MOESM5_ESM.pdf]

**Supplementary Table 4 Summary of abnormal behaviors exhibited by *Dock4* KO mice**

| Type of behaviors | Test | Result | Affected gender |
|---|---|---|---|
| Social behavior | Three-chamber | Lack of social novelty preference | M & F[1] |
| | Pup retrieval | Delayed pup retrieval | F (M not tested) |
| Language communication | Isolation-induced pup ultrasonic vocalizations | Increased number and duration of calls in pups during isolation | M & F |
| Repetitive behavior | Homecage observation and open field | Stereotyped circling in ~9% of female KO mice | F |
| Anxiety | Elevated Zero-maze | Low mobility in the open sections | M & F (M more severe) |
| Learning and Memory | Novel object recognition | Inability to distinguish familiar/novel objects | F |
| | Y-maze (spatial recognition) | Inability to distinguish familiar/novel arms | M |
| | Y-maze (spontaneous alternation) | Decreased alternation among arms and increased total entries | M |

[1]M: male; F: female
